# Mussel inspired 3D elastomer enabled rapid calvarial bone regeneration through recruiting more osteoprogenitors from the dura mater

**DOI:** 10.1093/rb/rbae059

**Published:** 2024-05-22

**Authors:** Xuqiao Wang, Chaoqun Ma, Xinchi Zhang, Pingping Yuan, Yujiao Wang, Mingdi Fu, Zheqian Zhang, Ruiying Shi, Na Wei, Juncheng Wang, Wei Wu

**Affiliations:** The College of Life Sciences, Northwest University, Xi'an, 710127, PR China; State Key Laboratory of Oral & Maxillofacial Reconstruction and Regeneration, National Clinical Research Center for Oral Diseases, Shaanxi Clinical Research Center for Oral Diseases, Department of Oral & Maxillofacial Surgery, School of Stomatology, The Fourth Military Medical University, Xi'an, 710032, PR China; State Key Laboratory of Oral & Maxillofacial Reconstruction and Regeneration, National Clinical Research Center for Oral Diseases, Shaanxi Clinical Research Center for Oral Diseases, Department of Oral & Maxillofacial Surgery, School of Stomatology, The Fourth Military Medical University, Xi'an, 710032, PR China; State Key Laboratory of Oral & Maxillofacial Reconstruction and Regeneration, National Clinical Research Center for Oral Diseases, Shaanxi Key Laboratory of Stomatology, Department of Prosthodontics, School of Stomatology, The Fourth Military Medical University, Xi'an, 710032, PR China; State Key Laboratory of Oral & Maxillofacial Reconstruction and Regeneration, National Clinical Research Center for Oral Diseases, Shaanxi Clinical Research Center for Oral Diseases, Department of Oral & Maxillofacial Surgery, School of Stomatology, The Fourth Military Medical University, Xi'an, 710032, PR China; State Key Laboratory of Oral & Maxillofacial Reconstruction and Regeneration, National Clinical Research Center for Oral Diseases, Shaanxi Clinical Research Center for Oral Diseases, Department of Oral & Maxillofacial Surgery, School of Stomatology, The Fourth Military Medical University, Xi'an, 710032, PR China; The College of Life Sciences, Northwest University, Xi'an, 710127, PR China; State Key Laboratory of Oral & Maxillofacial Reconstruction and Regeneration, National Clinical Research Center for Oral Diseases, Shaanxi Clinical Research Center for Oral Diseases, Department of Oral & Maxillofacial Surgery, School of Stomatology, The Fourth Military Medical University, Xi'an, 710032, PR China; State Key Laboratory of Oral & Maxillofacial Reconstruction and Regeneration, National Clinical Research Center for Oral Diseases, Shaanxi Clinical Research Center for Oral Diseases, Department of Oral & Maxillofacial Surgery, School of Stomatology, The Fourth Military Medical University, Xi'an, 710032, PR China; The College of Life Sciences, Northwest University, Xi'an, 710127, PR China; Institute of Stomatology, First Medical Center, Chinese PLA General Hospital, Beijing, 100853, PR China; State Key Laboratory of Oral & Maxillofacial Reconstruction and Regeneration, National Clinical Research Center for Oral Diseases, Shaanxi Clinical Research Center for Oral Diseases, Department of Oral & Maxillofacial Surgery, School of Stomatology, The Fourth Military Medical University, Xi'an, 710032, PR China

**Keywords:** 3D elastomer, macrophages, dural cells, polydopamine, bone healing

## Abstract

Currently, the successful healing of critical-sized calvarial bone defects remains a considerable challenge. The immune response plays a key role in regulating bone regeneration after material grafting. Previous studies mainly focused on the relationship between macrophages and bone marrow mesenchymal stem cells (BMSCs), while dural cells were recently found to play a vital role in the calvarial bone healing. In this study, a series of 3D elastomers with different proportions of polycaprolactone (PCL) and poly(glycerol sebacate) (PGS) were fabricated, which were further supplemented with polydopamine (PDA) coating. The physicochemical properties of the PCL/PGS and PCL/PGS/PDA grafts were measured, and then they were implanted as filling materials for 8 mm calvarial bone defects. The results showed that a matched and effective PDA interface formed on a well-proportioned elastomer, which effectively modulated the polarization of M2 macrophages and promoted the recruitment of dural cells to achieve full-thickness bone repair through both intramembranous and endochondral ossification. Single-cell RNA sequencing analysis revealed the predominance of dural cells during bone healing and their close relationship with macrophages. The findings illustrated that the crosstalk between dural cells and macrophages determined the vertical full-thickness bone repair for the first time, which may be the new target for designing bone grafts for calvarial bone healing.

## Graphical Abstract

**Figure:**
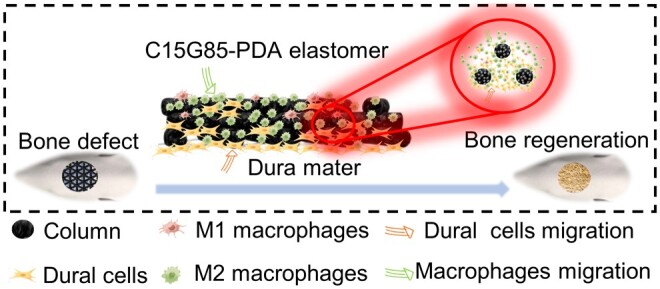

## Introduction

Calvarial repair remains a great clinical demand in surgical treatments, owing to the high occurrence of trauma, tumors and congenital bone deformities [1–4]. Currently, conventional bone grafts, including autografts, allografts and xenografts have been extensively employed in clinical settings [5, 6]. These treatments, however, are always associated with significant drawbacks, including insufficient bone tissue availability, donor site morbidity, immunogenicity and imprecise adaptation to the defect [7, 8]. Tissue engineering technology has provided an available alternative for *in situ* bone repair. However, there are still several unresolved issues, with one main challenge being the limited recruitment of endogenous stem cells (ESCs) [9–11]. Recent studies have mainly involved developing bioactive materials for more efficient bone repair by regulating M2 macrophages polarization and recruiting ESCs [12–16]. They aimed to create an ideal immune environment for osteogenesis through profound crosstalk between macrophages and ESCs that imitated natural state.

Mussels can adhere to wet solid surfaces, with phenylalanine (DOPA) and lysine-rich proteins identified as key substances for adhesion [17]. It was discovered that a biopolymer with the similar molecular structure to DOPA—polydopamine (PDA) has the same advantages as mussels. PDA could be easily deposited on almost all types of inorganic and organic matrices, including superhydrophobic surfaces [18–20]. Therefore, PDA, inspired by mussel adhesive proteins, has been recognized as an optimal surface modifier, owing to its high adhesiveness to various polymeric surfaces. Due to its exceptional biocompatibility, simple synthesis process, drug-loading capacity, as well as excellent immunomodulatory effects, PDA coating is widely employed to act as an interface between the implanted material and host body. Our previous study suggested that a porous 3D printed membrane, modified with PDA coating and used as a guided bone regeneration (GBR) membrane, enabled early and durable influx of M2 macrophages and promoted bone marrow mesenchymal stem cells (BMSCs) recruitment for efficient bone repair in 5 mm calvarial bone defects [13]. However, the GBR membrane exhibited the limited bone repair efficiency in 8 mm calvarial bone defects, which may be due to insufficient sources of ESCs (Supplementary Figure S1).

Bone regeneration in the skull defects depends not only on the ESCs from bone margins, but also on the ESCs from dura mater, which offer osteogenic precursor cells for bone healing [21–24]. The dura mater is closely associated with calvarial bone during development, leading to extensive interaction and regulation between them. It assists the formation of calvarial bone by supplying cytokines and osteoblast progenitor cells, as well as provides mechanical support for the development of cranial vault. Moreover, the dura mater is a major source of osteogenic cells during calvarial bone healing following injury. Previous studies have indicated that the dura mater had the capacity to form new bone or bone nodules [25–28]. *In vivo* studies where the dura mater was either removed or covered by a polytetrafluoroethylene membrane, the defect was filled with fibrous tissue with no bone island formation [22, 29, 30]. Therefore, adequate contact between the dura mater and the material may be a prerequisite for the effective calvarial bone healing. The limited efficacy of the PDA-modified GBR membrane in repairing an 8 mm calvarial bone defect may be attributed to its non-contact with the dura mater and inability to effectively recruit osteoprogenitors from the dura mater.

**Scheme 1.**
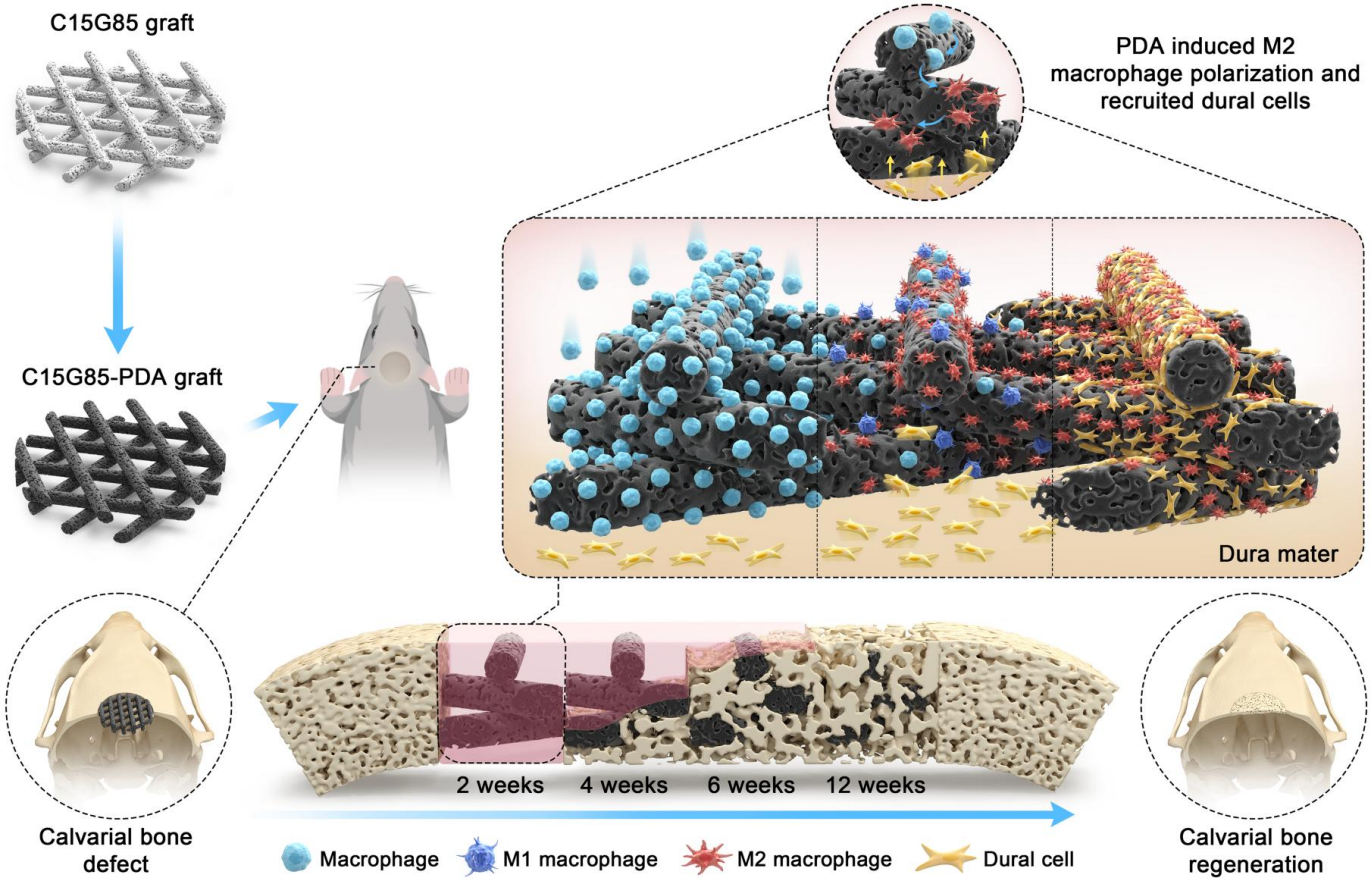
Schematic diagram illustrates that the early enrichment of M2 macrophages and dural cells inside the bone defect mediated by 3D elastomer contributes to calvarial bone healing.

In this study, the PDA-modified GBR membrane was improved as the PDA-modified filling scaffold of calvarial bone defect to fully contact with the dura mater. Unlike GBR membranes, the bone-filling materials required rational design regarding microstructure, filling rate and degradation rate, so that the influx of immune cells could be regulated to orchestrate stem cell recruitment. Therefore, the gradient porous and biodegradable materials with opening channels were fabricated by blending slow-degrading polymer polycaprolactone (PCL) and fast-degrading polymer poly(glycerol sebacate) (PGS) at various mass ratios *via* 3D printing, and subsequently modified with PDA as the calvarial bone-filling materials [31–33]. It aimed to explore whether immunomodulatory constructs may be designed to recruit more osteoprogenitors derived from the dura mater, and improve the healing efficiency of 8 mm calvarial bone defects. Combined with single-cell RNA analysis, it was found that the PDA interface with rational platform significantly orchestrated M2 macrophage polarization and dural cells recruitment, which achieved more osteogenesis for full-thickness regeneration of 8 mm calvarial bone defects (Scheme 1). Overall, it helped advance the field of endogenous bone regeneration for critical-sized defects.

## Materials and methods

### Materials

J&K Scientific supplied glycerol (high analytical purity of 99% grade) and sebacic acid (high analytical purity of 99% grade) for the purchase. Fuyu Chemical provided tetrahydrofuran (THF, ≥99.9% purity, anhydrous) for the purchase. Sigma-Aldrich supplied PCL (Mn 80 000 g/mol), while Ourchem provided salt particulates (NaCl, 99.5%). Dopamine hydrochloride (Dopa·HCl, 99%) was acquired from Sigma-Aldrich.

### Fabrication of the 3D-printed graft

A series of mass ratios of PGS prepolymer (pPGS) and PCL were dissolved in THF at a concentration of 15% (w/v). Fine-grade NaCl particles, ranging in size from 30 to 38 μm, were achieved through grinding and sifting. The polymer solution was subsequently blended with salt particles at a weight ratio of 1:2. After stirring the mixture for 24 h, it was transferred to a vacuum oven at 60°C and −0.08 MPa for an additional 24 h to ensure complete solvent removal. The formulated PCL/PGS composite ink was utilized in the 3D printing process to create 9-layered and 3-layered constructs. The printing occurred at 60°C under a pressure of 0.25 MPa, using the 3D printing system (PC Printer BR151S; Particle Cloud Biotechnology, Co., Xi’an, China).

The designated printing parameters are outlined as follows: the nozzle diameter is 0.5 mm, with a layer height set to 0.3 mm. The angle between consecutive layers is fixed at 60°, and the infill density is 25%. Additionally, the extrusion rate is maintained at 1 mm/s. Subsequently, the printed constructs underwent thermal crosslinking in a vacuum oven. The process involved 12 h at 80°C under −0.05 MPa and an additional 24 h at 150°C under −0.1 MPa. To eliminate the NaCl particles, they were removed by soaking the constructs in ddH2O for 24 h, with water changes every 8 h. The C0G100, C15G85 and C30G70 (PGS: PCL = 100:0, 85:15 and 70:30, respectively) grafts were obtained. To enhance the biological properties of the material, C0G100, C15G85 and C30G70 grafts were submerged in a 10 mM Tris–HCl buffer solution with a pH of 8.5 for 24 h to obtain C0G100-PDA, C15G85-PDA and C30G70-PDA grafts, respectively. Unbound PDA was removed by washing several times with ddH_2_O.

### Characterization of 3D-printed porous elastomeric grafts

#### Morphology of the grafts

The different samples prepared were attached to aluminum stubs and then coated with a layer of gold using sputter coating. Scanning electron microscopy (SEM, S-4800, Hitachi) was employed for sample analysis. For every individual sample, three regions were arbitrarily chosen for visual examination, and this process was repeated for three samples in each group. The superficial and transverse structures of the created grafts were scrutinized at different magnification levels. The analysis of surface porosity was conducted employing the ImageJ software developed by the National Institutes of Health.

#### X-ray photoelectron spectroscopy (XPS)

XPS was employed to acquire spectra for different samples using the Thermo SCIENTIFIC ESCALAB Xi+ system. The analysis aimed to determine atomic compositions. Data collection utilized a monochromatic X-ray source (Al Kα). The obtained XPS spectra were subjected to peak-fitting using Advantage processing software from Thermo Fisher Scientific, USA.

#### Mechanical properties

The mechanical properties of the C0G100, C15G85, C30G70, C0G100-PDA, C15G85-PDA and C30G70-PDA grafts were assessed using a tensile testing machine (Sansi, UTM4103, China), which conducted both tensile and compression testing. For the tensile tests, strips measuring 20 mm × 15 mm × 1 mm were used, and the testing occurred at a rate of 2 mm/min. Cuboid samples sized 10 mm × 10 mm × 3 mm were employed for compression testing, conducted at a rate of 2 mm/min. The tensile stress and strain, as well as the compressive stress and strain at 75% compression, were derived from the tests.

#### Water contact angles

All samples were placed on glass slides and a 4 μl droplet of ddH2O was deposited on the sample surfaces. Water contact angles (WCA) between the droplets and the surface of the samples were quantified utilizing a drop shape analysis system (Krüss, EASY DROP K100, Germany), and the results were processed with image analysis using DSA 1.90.0.2 software at room temperature.

#### The 4D-morphing and instantly fixable capability

During heat curing, the constructs could be molded into almost any shape. A simple evaluation of 4D graft shaping on silicon tubes was conducted. Using the unique elasticity of the PCL/PGS material, a circular C15G85-PDA graft with a diameter of 6 cm was filled into a 5.8 cm 3D printed human skull defect model to observe the instantly fixable capability. Meanwhile, the sheet material of C15G85 was shaped into a tubular material and the shape was recorded at 0, 1, 2 and 4 weeks.

### Cell experiments

#### Biocompatibility evaluation

A series of mass ratios of PGS and PCL were dissolved in THF at a concentration of 25% (w/v) to obtain a polymer solution. The 15 mm cell-climbing slices were immersed in the solution to prepare polymer membranes tightly bound to the cell-climbing slices, and then the polymer membranes were treated according to the method described in Section ‘Fabrication of the 3D-printed graft’ to obtain the 0G100, C15G85, C30G70, C0G100-PDA, C15G85-PDA and C30G70-PDA membrane. Rat bone mesenchymal stem cells (BMSCs) were seeded on the membrane surface in 24-well plates at a concentration of 2 × 10^4^ cells/well. The biocompatibility of the grafts was assessed on days 1, 3 and 5 using the Live/Dead Staining Kit (Yeasen, Shanghai, China). Cell proliferation of each group (untreated medium served as the control group) was assessed on days 1, 3 and 5 using the CCK-8 assay (Dojindo Kagaku, Japan).

#### *In vitro* immunomodulation effect of the grafts to the macrophages

RAW 264.7 cells were seeded on the membrane surface of C15G85, C0G100-PDA, C15G85-PDA and C30G70-PDA groups in 24-well plates plated with (2 × 10^4^ cells per well). The macrophage polarization was evaluated by immunofluorescence staining after 48 h. Briefly, RAW 264.7 cells were treated with 3% glutaraldehyde, 0.5% Triton X-100 solution and 5% bovine serum albumin solution. The samples were, respectively, double-labeled with CD68/iNOS and CD68/CD206 primary antibodies at 4°C overnight, and then anti-mouse/rabbit secondary antibodies were applied for 1 h incubation in the dark. The anti-fluorescence quencher was used to seal the sections (with DAPI). Details of primary antibodies and secondary antibodies are listed in Supplementary Tables S1 and S2. After staining, the samples were observed by a confocal laser scanning microscope (FV 1000, Olympus, Japan).

### Animal grouping and surgery

The surgical interventions were carried out on male SD rats aged 4 weeks, with weights ranging from 100 to 150 g. The rats were procured from the Animal Research Center at Fourth Military Medical University, Xi'an, China, and were acclimatized to an SPF environment. Approval for all animal procedures was obtained from the Institutional Animal Care Committee of the Fourth Military Medical University (2022-kq-016), ensuring compliance with the Guide for Care and Use of Laboratory Animals.

Under isoflurane anesthesia (4%), all surgical procedures were conducted. Surgical sites underwent sterilization applying a sophisticated iodine solution following hair removal using a clipper. To formulate the calvarial defect model, a cranial defect of 8 mm in diameter was crafted at the central locus of the calvarium, employing a dental trephine. Rats from the C15G85-PDA group were euthanized, and the tissues from the defective regions were extracted for single-cell collection at 2 weeks. In addition, the rats of the C15G85, C0G100-PDA, C15G85-PDA and C30G70-PDA groups were sacrificed and the regenerative tissues were harvested and frozen sections were used for immunofluorescent staining at 2 weeks.

The rats of the sham, C0G100, C15G85, C30G70 groups were sacrificed at 6 and 12 weeks, while the C0G100-PDA, C15G85-PDA and C30G70-PDA groups were sacrificed at 4, 6 and 12 weeks after surgery and the calvarial specimens were procured for subsequent evaluations.

To examine whether macrophages participated in bone healing conducted by C15G85-PDA graft, 8 mm calvarial bone defects were generated by the above method, and rats were arranged into two groups: the C15G85-PDA + Clodronate group was treated with clodronate liposomes (The Netherlands) to deplete macrophages, whereas the C15G85-PDA + PBS group was treated with PBS liposomes (The Netherlands). All the liposomes were given locally *via* injection every 2 days after graft implantation at the first 2 weeks, and subsequently once a week. The rats were sacrificed at 2, 4, 6 and 12 weeks.

*In vivo* subcutaneous graft implantation models were established in rats to assess the degradation properties of the grafts. In brief, SD rats were anesthetized following the previously described anesthesia procedure. On the back, six areas symmetrically located along the spine (three on each side) were shaved and disinfected with iodophor. To establish six subcutaneous pockets, 1-cm-long vertical incisions were made, and grafts of each group were carefully inserted. Rats were sacrificed at 2, 4, 6 and 12 weeks after surgery, and the harvested grafts were gathered for further analysis.

### Micro-CT analysis

The calvarial samples were collected and immersed in 4% paraformaldehyde for a duration of 48 h. The fixed samples underwent scanning using a Siemens Micro-CT system (Siemens, Germany) at a resolution of 10 μm. For 3D reconstruction and analysis, Micro-CT software (Inveon Research Workplace, Siemens, Germany) was employed. A defined cylindrical space, delineating the region of interest, was established to assess both the volume of bone and tissue, enabling the computation of BV/TV (bone volume/total volume) and Tb.N (Trabecular Number).

### Histological analysis

Samples extracted from the calvarial defect area at 4, 6 and 12 weeks were subjected to decalcification using 10% ethylene-diamine tetra-acetic acid for 6 weeks. Following that, a systematic dehydration process using a series of graded alcohol concentrations was implemented, culminating in the embedding of the specimens in paraffin. Successive sections, each with a thickness of 4 μm, were procured from the region of the defect and underwent HE staining, Safranin-O staining and Masson's trichrome staining to assess the generation of new bone tissue and the presence of residual graft material. For samples from subcutaneous graft implantation, collected at 2, 4, 6 and 12 weeks after surgery, they were embedded in OCT Embedding Medium (Sakura Finetek, CA, USA) for fresh frozen sections. Subsequently, the samples were processed for H&E staining according to standard methods. Digital photography of the stained specimens was conducted, and they were viewed using Digital Slide Scanners (Pannoramic MIDI II, 3DHISTECH).

### Quantification of PGS/PCL residuals in grafts

To quantify the graft residual area in each group, the polarized H&E images in each group at 2, 4, 6 and 12 weeks were used according to the methods reported previously [34]. The PCL residuals were identified by specific white birefringence under polarized light, whereas PGS showed no birefringence. The PGS residual appeared as a slightly stained porous structure under bright field. The areas of PCL residuals and the residual graft material (PCL + PGS) were measured using ImageJ software.

### Detection of graft degradation products

The C15G85-PDA graft underwent an enzymolysis experiment for 21 days at 40°C, using hyphal lipase B with a concentration of 100 U/mL (Beijing Gaorisen Technology Co., LTD, China). The components of the degradation solution and the silanized derivatives were then detected and analyzed using an Agilent 8860-5977B gas chromatography–mass spectrometer. The silanized derivatives were manually reduced to the active hydrogen to obtain the final products.

### Immunofluorescent staining

The surrounding bone tissue of samples from the defect area at 2 weeks was removed, and the residual soft tissues containing grafts were embedded with OCT and promptly cryopreserved at −80°C. The specimens were subsequently sectioned into slices with a thickness of 5 μm. The sections underwent fixation with ice-cold acetone for a duration of 20 min, succeeded by three rinses with PBS. Following that, a 0.3% H_2_O_2_ treatment lasting 15 min was applied, succeeded by incubation in a blocking solution. The samples received overnight treatment with primary antibodies at a temperature of 4°C, encompassing CD68 as a macrophage marker, CD206 as an M2 macrophage marker, iNOS as an M1 macrophage marker, Alpl and Mgp as dural cell markers. After three PBS rinses, the samples underwent incubation with fluorescent secondary antibodies for a duration of 1 h at 37°C. Details of primary anti-bodies and secondary antibodies are listed in Supplementary Tables S1 and S2. After incubation, the anti-fluorescence quencher was used to seal the sections (with DAPI). After staining, observations of the samples were conducted utilizing a confocal laser scanning microscope (FV 1000, Olympus, Japan) and were quantified using ImageJ software.

### Immunohistochemistry staining

In brief, antigen retrieval was performed using pepsin (Maixin Biological Technology Development Company, China) after deparaffinization and rehydration. The endogenous peroxidase was neutralized using endogenous peroxidase blocking solution and subjected to an overnight incubation at 4°C, the specimens were treated with primary antibodies targeting COL2 and HIF-1α (Supplementary Table S1). After three PBS rinses, slides underwent treatment with ready-to-use secondary antibodies conjugated with horseradish peroxidase (Zhongshan Golden Bridge Biotechnology, China). Incubation occurred at 37°C for a duration of 1 h, and the ensuing reaction was disclosed through the utilization of DAB chromogenic solution, succeeded by counterstaining with hematoxylin. The specimens, after staining, were digitally photographed and examined using Digital Slide Scanners (Pannoramic MIDI II, 3DHISTECH).

### Single-cell RNA sequencing and data analysis

#### Single-cell dissociation

Soft tissue samples with grafts from the calvarial defect area were collected after 2 weeks and underwent three rounds of PBS rinsing. Subsequently, the excised tissues were maintained in MACS Tissue Storage Solution (Miltenyi Biotec) until the processing stage. The processing of the tissue samples followed the description provided below. The samples underwent an initial PBS wash, followed by mincing into small fragments (approximately 1 mm^3^) on ice, and then an enzymatic digestion was performed using 200 μl Enzyme H, 100 μl Enzyme R and 25 μl Enzyme A for 30 min at 37°C with continuous agitation. Following the digestion process, the samples were filtered through a 70 µm cell strainer and then subjected to centrifugation at 300 g for 5 min. Upon removal of the supernatant, the pelleted cells were resuspended in red blood cell lysis buffer (Miltenyi Biotec) for the purpose of lysing red blood cells. Following rinsing with PBS containing 0.04% BSA, the cell pellets were re-suspended in PBS containing 0.04% BSA and re-strained through a 35 μm cell strainer. The dissociated individual cells were subsequently labeled with AO/PI for the evaluation of viability utilizing the Countstar Fluorescence Cell Analyzer. The suspension of single cells underwent additional enrichment using a MACS Dead Cell Removal Kit (Miltenyi Biotec).

The analysis of scRNA-seq (single-cell RNA sequencing) data was conducted by NovelBio Co., Ltd utilizing the NovelBrain Cloud Analysis Platform, accessible at www.novelbrain.com. The fastp with default parameters was utilized to filter out adapter sequences and eliminate low-quality reads, resulting in the generation of clean data. Subsequently, the feature-barcode matrices were generated by aligning reads to the mouse genome (mm10 Ensemble: version 100) using CellRanger v3.1.0. Downsampling analysis was conducted on the sequenced samples based on the mapped barcoded reads per cell for each sample. Ultimately, this process led to the creation of the aggregated matrix. Cells that exhibited expression in more than 200 genes and a mitochondria UMI rate below 20% successfully passed the quality filtering criteria, with mitochondria genes subsequently excluded from the expression table.

#### Gene ontology analysis

Conducting gene ontology (GO) analysis aimed at shedding light on the biological implications of both marker genes and differentially expressed genes. The GO annotations were acquired by downloading data from NCBI, UniProt and the Gene Ontology. To identify significant GO categories, Fisher’s exact test was employed and the FDR was subsequently utilized for the correction of *P*-values.

#### Pseudo-time analysis

The single-cell trajectories analysis was conducted using Monocle2 (http://cole-trapnell-lab.github.io/monocle-release) with DDR-Tree and default parameters. Prior to initiating the Monocle analysis, marker genes derived from the Seurat clustering outcome were chosen, utilizing raw expression counts from cells that had successfully passed the filtering process. Using the results of the pseudo-time analysis, the Branch Expression Analysis Modeling (BEAM Analysis) was employed to conduct gene analysis for determining branch fate. Additionally, RNA velocity analysis was conducted using the scvelo Python package (version 0.1.25).

#### Cell communication analysis

Facilitating a systematic examination of cell–cell communication molecules, the CellPhoneDB was utilized to implement a cell communication analysis. This publicly available repository encompasses information on ligands, receptors and their interactions. Proteins localized in the membrane, secreted proteins and peripheral proteins of the cluster at different time points were annotated. The calculation of significant mean and cell communication significance (*P *<* *0.05) was performed using the interaction data and the normalized cell–matrix obtained through Seurat Normalization.

### Statistical analysis

The replicate experiments encompassed biological replicates conducted on at least three instances. Data are presented as mean ± SD and underwent statistical comparison using unpaired *t*-test, ANOVA or two-way ANOVA, employing Prism 9.0 software (GraphPad Prism). Statistical significance was determined at *P *<* *0.05. Notation for significance levels: *P <* *0.05; **P <* *0.01; ***P <* *0.001; ****P <* *0.0001.

## Results

### 3D Printing enabled the tunability of microstructure, mechanical strength and PDA coating

**Figure 1.**
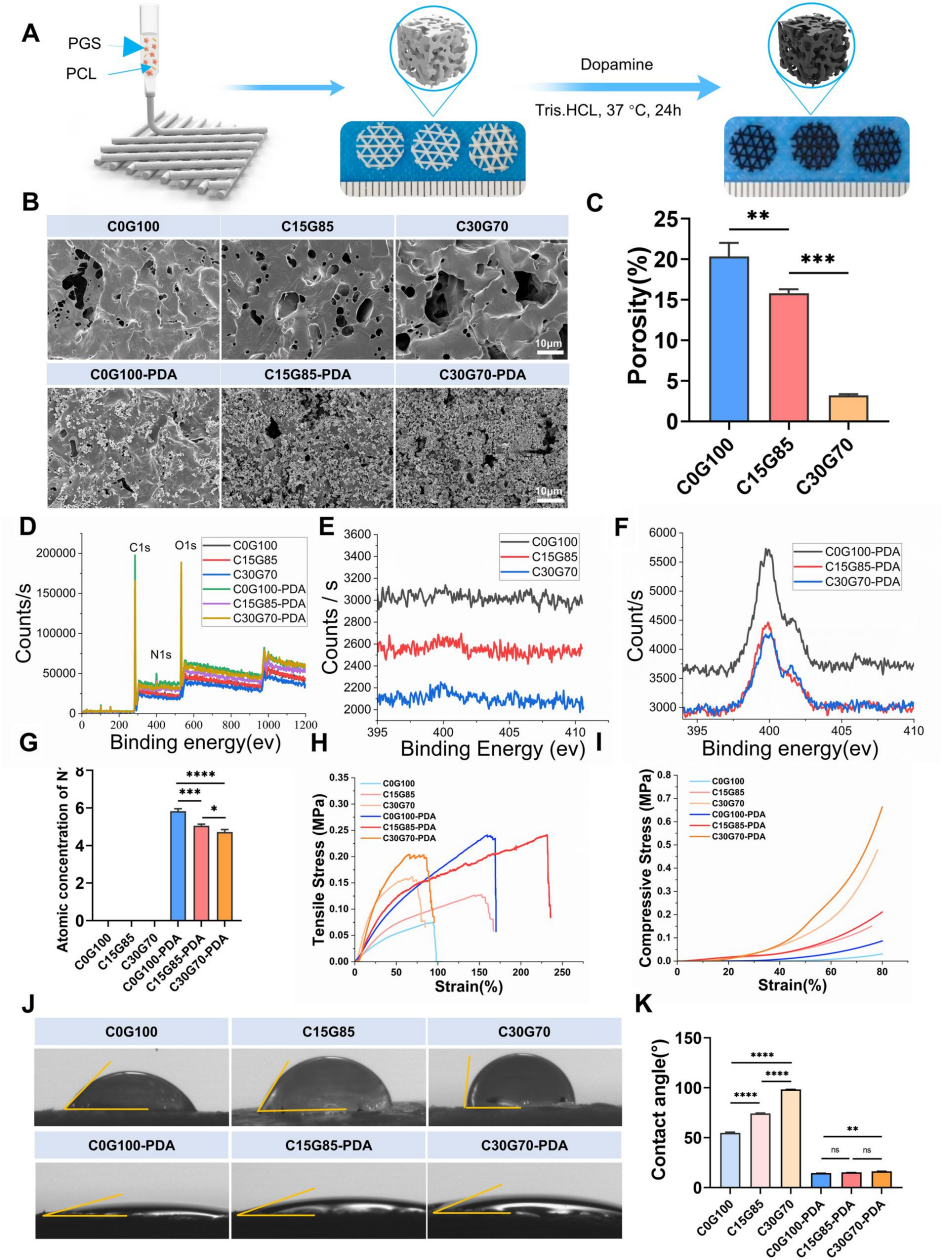
Characterization of 3D porous elastomers. (**A**) Schematic diagram of fabrication process. (**B**) SEM examination showed microstructures of 3D porous elastomers. (**C**) Surface porosity of 3D porous elastomers (*n* = 3). (**D**) XPS total spectrum of 3D porous elastomers. (**E**). XPS spectrum for N 1 s of the C0G100, C15G85 and C30G70 groups. (**F**). XPS spectrum for N 1 s of the C0G100-PDA, C15G85-PDA and C30G70-PDA groups. (**G**) Quantitative analysis of XPS (*n* = 3). (**H**) Tensile stress–strain curves of 3D porous elastomers. (**I**) Compressive stress–strain curves of 3D porous elastomers. (**J**) The hydrophilicity of 3D porous elastomers was detected by water contact angle. (**K**) Quantitative comparison of WCA (*n* = 3). Data are presented as mean ± SD. ns, *P *>* *0.05; *P <* *0.05; **P <* *0.01; ***P <* *0.001; ****P <* *0.0001.

The C0G100, C15G85 and C30G70 grafts were fabricated by using 3D printing technology, with filling rate of 25%. C0G100, C15G85 and C30G70 grafts were coated with PDA to produce C0G100-PDA, C15G85-PDA and C30G70-PDA grafts, respectively (Figure 1A). SEM showed that the printed PCL underwent melting during thermal crosslinking, which resulted in decreased surface porosity on columns as PCL content increased (C0G100, 20.36 ± 1.67%; C15G85, 15.82 ± 0.47%; C30G70, 3.19 ± 0.18%) (Figure 1B and C and Supplementary Figure S2). PDA nanoparticles were distributed on the column surface, and the more adherent PDA nanoparticles presented with increasing micropores and PGS content (Figure 1B and Supplementary Figure S2). As shown in Figure 1D–F, different N1s peaks were observed in the C0G100, C15G85, C30G70, C0G100-PDA, C15G85-PDA and C30G70-PDA groups, which was produced by the secondary amine (NH) and minor primary amino (NH_2_) groups after PDA coating. Atomic concentration of N1s suggested missing N1s in the C0G100, C15G85 and C30G70 groups, while the C0G100-PDA, C15G85-PDA and C30G70-PDA groups presented different amounts of N1s, which decreased with increasing PCL content (C0G100-PDA, 5.83 ± 0.13%; C15G85-PDA, 5.05 ± 0.09%; C30G70-PDA, 4.72 ± 0.14%) (Figure 1G). The results were consistent with variant amounts of PDA in the different groups appeared in SEM images.

The mechanical properties also varied significantly with the change of PCL content. Both the tensile and compressive properties increased with increasing PCL content, and further improvements were observed after the PDA coating (Figure 1H and I). The tensile moduli of 0G100, C15G85, C30G70, C0G100-PDA, C15G85-PDA and C30G70-PDA grafts were 0.12 ± 0.00, 0.17 ± 0.00, 0.59 ± 0.02, 0.23 ± 0.01, 0.30 ± 0.00 and 0.62 ± 0.00 MPa, respectively (Supplementary Figure S3A). The compressive moduli of 0G100, C15G85, C30G70, C0G100-PDA, C15G85-PDA and C30G70-PDA grafts were 0.07 ± 0.00, 0.30 ± 0.03, 1.32 ± 0.02, 0.15 ± 0.02, 0.41 ± 0.03 and 1.40 ± 0.02 MPa, respectively (Supplementary Figure S3B). The WCA of the constructs were measured to evaluate their hydrophilic properties. The WCA also increased with increasing PCL content, and WCA of the C0G100-PDA, C15G85-PDA and C30G70-PDA groups (14.93 ± 0.15°, 15.26 ± 0.15° and 16.43 ± 0.32°, respectively) were significantly smaller than those of the C0G100, C15G85 and C30G70 groups (54.93 ± 0.63°, 74.33 ± 0.47° and 98.45 ± 0.45°, respectively) (Figure 1J and K). These results indicated that the hydrophilicity of constructs was improved by PDA coating and decreased with increasing PCL content.

What’s more, 4D shaping of the C15G85 membrane around silicon tube was performed and morphology was well maintained before and after PDA coating (Supplementary Figure S4A). The 3D graft (6.0 cm in diameter) was instantly fixed to the defect (5.8 cm in diameter) of a 3D printed human skull model without metal plates (Supplementary Figure S4B), suggested a simplified surgical procedure in clinical translation. Meanwhile, the shape of the C15G85-PDA membrane remained stable over time after it has been transformed into a specific model (Supplementary Figure S5A and B).

### Biodegradability of 3D elastomers and the stability of PDA interface *in vivo*

In addition to the variations in graft microstructure, mechanical strength and PDA content, different ratios of PCL and PGS also resulted in the variant degradation rates, which could potentially impact the stability of PDA interface. The grafts composed of PCL, PGS and PDA have good biocompatibility (Supplementary Figure S6) and biosafe degradation components (Supplementary Supplementary Figure S7). To evaluate their degradation rates, the grafts were implanted into dorsal subcutaneous pouches of rats. After subcutaneous implantation for 2 weeks, the graft material of the C0G100 group completely degraded (no residue found), while the remaining grafts in the other groups were taken out for further study. H&E staining results suggested that the graft material of the C15G85 group was more stable than that of the C30G70 group (Supplementary Figure S8A and C), despite the higher concentration of PCL in the latter group (Supplementary Figure S8B). The results of polarized light microscopy showed that PCL was distributed in scattered dots in the column, and the addition of more PCL could not contribute to stable degradation (Supplementary Figure S8A).

**Figure 2.**
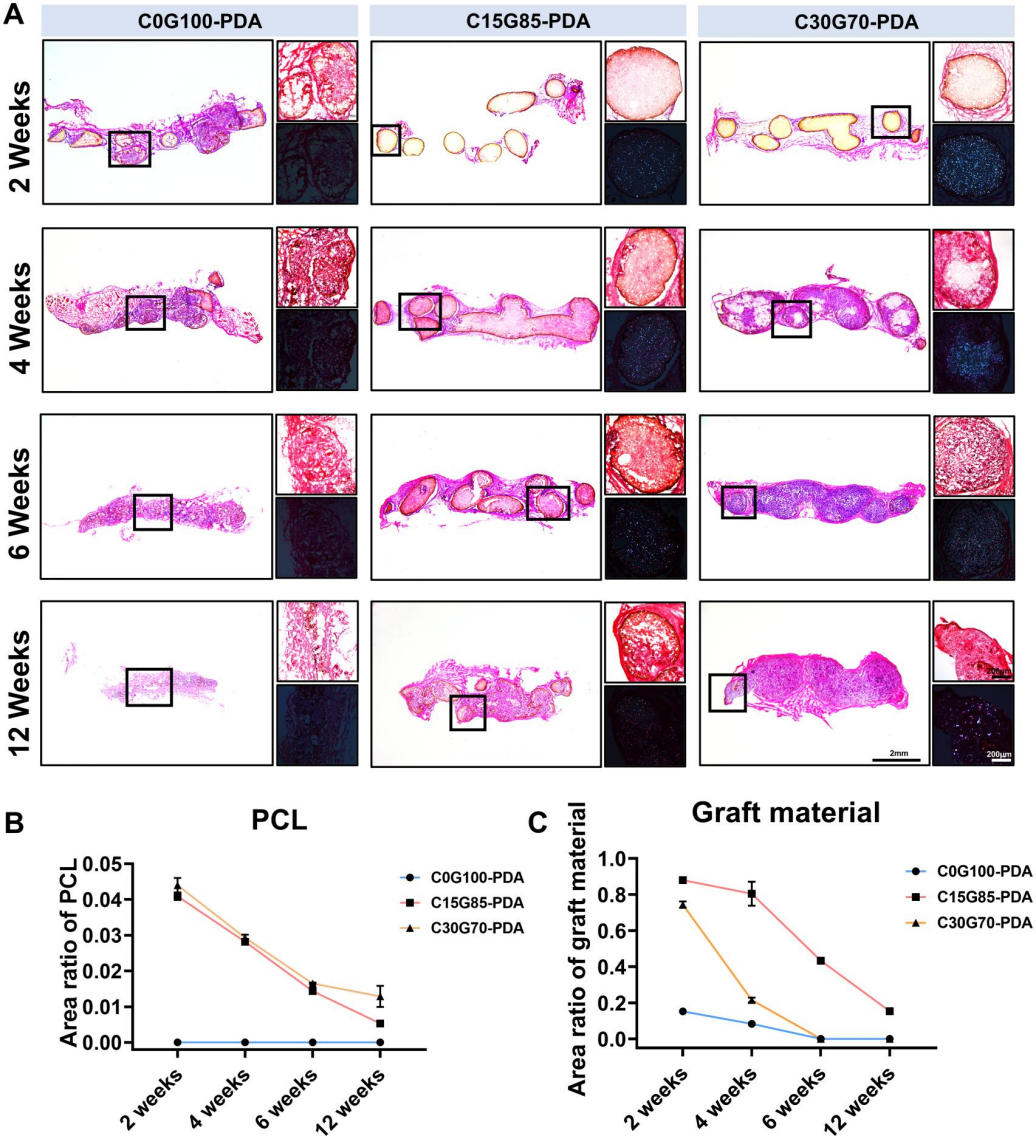
Biodegradability of 3D elastomers and the stability of PDA interface *in vivo* (**A**) H&E staining visualized degradation patterns of critical bone defects in the C0G100-PDA, C15G85-PDA and C30G70-PDA groups at 2, 4, 6 and 12 weeks. Quantitative comparison of remaining area of (**B**) PCL and (**C**) graft material (*n* = 3). Data are presented as mean ± SD. ns, *P *>* *0.05; *P <* *0.05; **P <* *0.01; ***P <* *0.001; ****P <* *0.0001.

The graft material of each group showed a similar degradation trend after PDA coating. The graft material of the C15G85-PDA group remained more stable than those of the C0G100-PDA and C30G70-PDA groups (Figure 2A and B), despite the C30G70-PDA group exhibited a higher concentration of PCL (Figure 2C). Meanwhile, the PDA interface of the C15G85-PDA group exhibited greater stability compared to the other groups within 12 weeks, during which the PDA coating obviously in turn delayed the degradation rate of grafts at the same time point (Figure 2A). The disintegration of PDA interface in the C0G100-PDA group, C15G85-PDA group and C30G70-PDA group started at 2, 4 and 6 weeks, respectively. The disintegrating PDA nanoparticles were dispersed in the surrounding tissues, especially inside the columns, which may affect the function of PDA. Moreover, the results of the gross appearance at 12 weeks remained consistent with the H&E results, which confirmed that the degradation of the C15G85-PDA group was the most stable (Supplementary Figure S9). Therefore, the ratio of PCL and PGS was important for the stability of PDA interface. Only C15G85-PDA grafts exhibited a stable and gradient degradation rate through 12 weeks, which may offer a stable platform for PDA to exert immunomodulatory effects.

### 3D elastomer enabled rapid calvarial bone regeneration by both endochondral and intramembranous ossification

**Figure 3.**
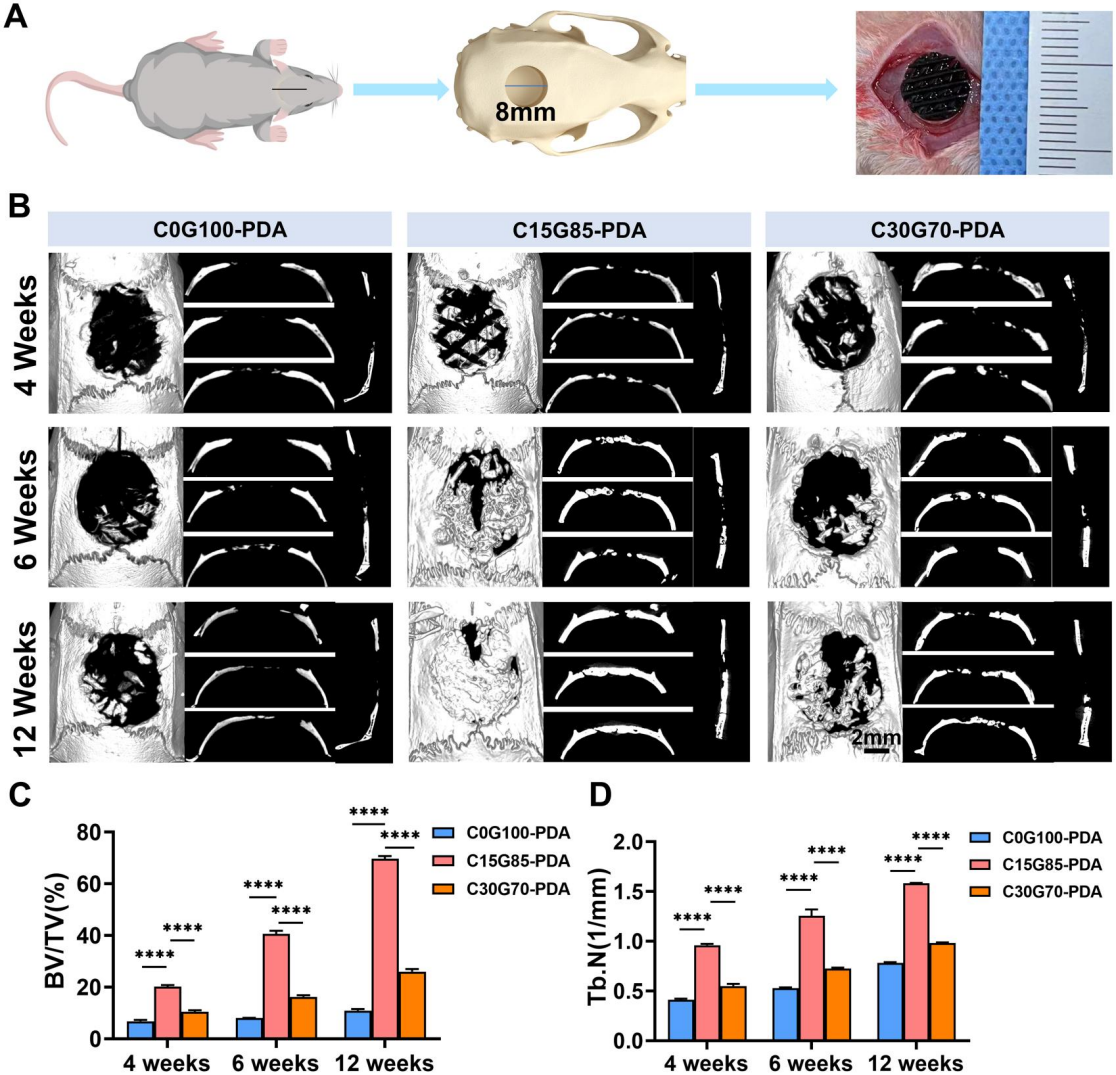
3D elastomer enabled rapid calvarial bone regeneration. (**A**) Schematic diagram of 3D porous elastomers’ application in rat calvarial defects. (**B**) 3D reconstruction and coronal/sagittal plane analysis of the defects in the C0G100-PDA, C15G85-PDA and C30G70-PDA groups at 4, 6 and 12 weeks. Quantitative analysis of (**C**) BV/TV and (**D**) Tb.N at 4, 6 and 12 weeks of the different groups (*n* = 5). Data are presented as mean ± SD. ns, *P *>* *0.05; *P <* *0.05; **P <* *0.01; ***P <* *0.001; ****P <* *0.0001.

**Figure 4.**
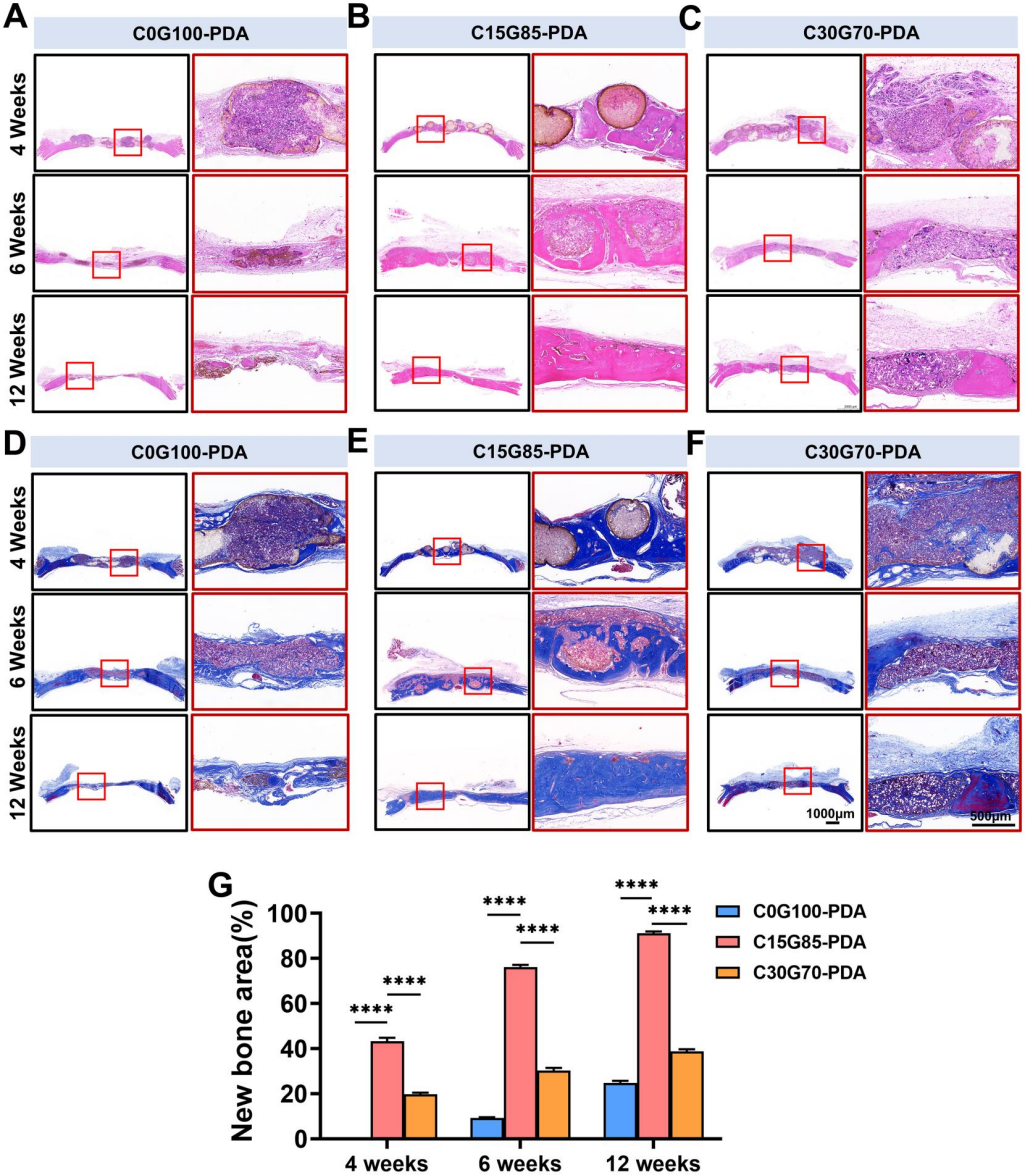
Examining of the bone regeneration. H&E staining showed the repair effects of critical bone defects at (**A**) 4, (**B**) 6 and (**C**) 12 weeks in the different groups. Masson’s trichrome staining assessed the collagen deposition of critical bone defects at (**D**) 4, (**E**) 6 and (**F**) 12 weeks in the different groups. (**G**) Quantitative comparison of new bone area (*n* = 5). Data are presented as mean ± SD. ns, *P *>* *0.05; *P <* *0.05; **P <* *0.01; ***P <* *0.001; ****P <* *0.0001.

To evaluate the bone healing potential mediated by PDA interface in different groups *in vivo*, 3D-printed grafts were implanted in rat 8 mm calvarial bone defects (Figure 3A). In detail, circular defects (diameter = 8 mm) were created and filled with 8 mm C0G100, C15G85, C30G70, C0G100-PDA, C15G85-PDA and C30G70-PDA grafts, the defects without grafts were set as the sham group. All rats survived after the surgical procedure, and no infections were found. The samples were harvested to quantify the new bone using Micro-CT scanning. As shown in Supplementary Figure S10A, there was little new bone formation at 6 weeks in the sham, C0G100, C15G85 and C30G70 groups (BT/TV: 2.11 ± 0.34%, 2.01 ± 0.08%, 2.49 ± 0.14% and 2.55 ± 0.06%, respectively), and only a small amount of new bone formation was observed until 12 weeks (BT/TV, 8.60 ± 0.45%, 10.88 ± 0.59%, 13.31 ± 0.52% and 11.78 ± 0.72%, respectively) (Supplementary Figure S10B). Compared with these results, more new bone formation was observed in the C0G100-PDA, C15G85-PDA and C30G70-PDA groups at 4 weeks after implantation, especially in the C15G85-PDA group (BV/TV, C0G100-PDA, 6.74 ± 0.57%; C15G85-PDA, 20.19 ± 0.58%; C30G70-PDA, 10.45 ± 0.57%) (Figure 3B and C). The results suggested that PDA played an important role in bone regeneration and the regeneration efficiency mediated by PDA in each group was different. Moreover, the new bone regenerated in a multi-point pattern and appeared disconnected with the host bone at 4 weeks, especially in the C15G85-PDA group (Figure 3B). Together with previous reports [25, 30], these results suggested that the main cellular source of new bone may be dural cells. The results of H&E staining were consistent with those of Micro-CT, which presented the new bone growing from the dura mater side and surrounding the complete PDA interface in the C15G85-PDA group, while the other two groups presented less new bone and the disintegrating PDA interfaces at 4 weeks (Figure 4A–C). The findings demonstrated that the C0G100-PDA and C30G70-PDA grafts assisted bone healing to a certain extent, while the C15G85-PDA graft contributed to remarkable bone regeneration, covering almost two-thirds of the entire defect at 6 weeks (BV/TV, C0G100-PDA, 8.01 ± 0.07%; C15G85-PDA, 40.59 ± 1.19%; C30G70-PDA, 16.25 ± 0.58%) (Figure 3B and C). The H&E staining showed that the newly formed bone infiltrated into the material columns of graft, forming ‘C’-shaped bony rings surrounding the PDA interface of the C15G85-PDA group at 6 weeks (Figure 4B). Consistently, the C0G100-PDA and C30G70-PDA grafts failed to fully repair the defects, while the C15G85-PDA graft completed the regeneration of the entire defect at 12 weeks (BV/TV, C0G100-PDA, 10.88 ± 0.59%; C15G85-PDA, 69.73 ± 0.99%; C30G70-PDA, 25.98 ± 0.99%) (Figure 3B and C). Meanwhile, the value of Tb.N in the different groups showed a trend consistent with BV/TV (Figure 3D). During this process, the degradation pattern of graft and the distribution of PDA were consistent with the trend of the subcutaneously implanted material (Figures 2A and 4A). These results indicated that the matched PDA interface was vital for the newly formed bone. In addition to the increased bone mass, the histological structure and collagen component of new bone tissue were also assessed. The presence of the newly formed bone was further confirmed by Masson’s Trichrome staining (Figure 4D–F and Supplementary Figure S11B). Taken together, the results showed that the full-thickness regeneration of calvarial bone defect was achieved by the C15G85-PDA graft.

The type of new bone formation during bone repair was studied using Safranin-O/fast green staining and immunohistochemical techniques. The results indicated that the calcified bone formed in channels, while the cartilage-like tissues formed inside the columns of C15G85-PDA group at 6 weeks (Supplementary Figure S12A and C). Moreover, the immunohistochemical staining exhibited a higher intensity of HIF-1α (hypoxia-inducible factor 1α) inside the columns compared to the staining intensity among the columns, suggesting a relatively lower concentration of oxygen inside the columns (Supplementary Figure S12B). In conclusion, the rapid bone regeneration in the C15G85-PDA group could be due to the simultaneous existence of both intramembranous and endochondral ossification from dural cells.

### Immunomodulatory effects of PDA-coated grafts *in vitro* and *in vivo*

After confirming the positive effect of the C15G85-PDA graft on bone regeneration, the study proceeded to examine the macrophage polarization *in vitro*. Immunofluorescent staining showed a higher proportion of iNOS+ cells in the C15G85 group compared to other PDA-coated groups (Supplementary Figure S13A and B). In contrast, the proportion of CD206+ cells in the C15G85 group was lower than that in other PDA-coated groups. Moreover, the proportion of CD206+ cells decreased with increasing PCL content, which may be related to the amount of PDA (Figure 1G and Supplementary Figure S13A and C). These results indicated that the PDA coating polarized macrophages to an M2-like phenotype *in vitro*.

**Figure 5.**
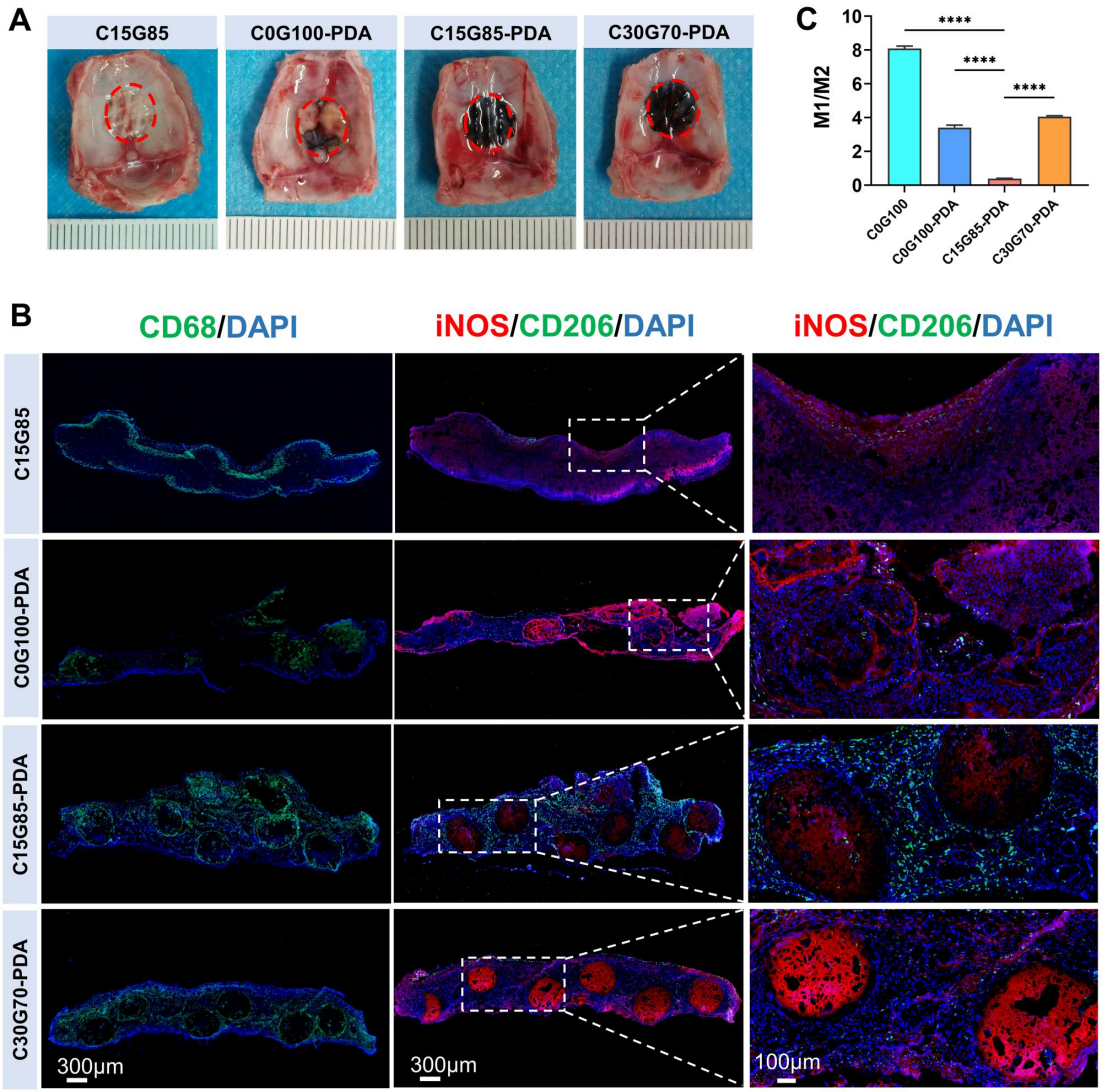
Early pro-healing microenvironment in calvarial bone defect. (**A**) The interior views of 3D porous elastomers after 2 weeks. Red circle marks boundary of the 8 mm rat calvarial defect. (**B**) Representative immunofluorescence showed M1 and M2 macrophage polarization in the C15G85, C0G100-PDA, C15G85-PDA and C30G70-PDA groups at 2 weeks. (**C**) Quantitative comparison of M1/M2 (*n* = 5). Data are presented as mean ± SD. ns, *P *>* *0.05; *P <* *0.05; **P <* *0.01; ***P <* *0.001; ****P <* *0.0001.

To verify the immunoregulation effect of the grafts *in vivo*, the C15G85, C0G100-PDA, C15G85-PDA and C30G70-PDA grafts were implanted in the calvarial bone defects. The grafts completely fused with the host bone at 2 weeks, and the tissues located within the red circles were entirely harvested for immunofluorescence staining (Figure 5A and B). The CD68 staining revealed that macrophages were predominantly derived from the skin over the defect. As indicated in Figure 5B, the regenerative tissue of the C15G85-PDA group was associated with more CD206+ cells and fewer iNOS+ cells, and a CD206+ cells were aggregating band formed around the PDA interface of the columns, while the regenerative tissues of the C15G85, C0G100-PDA and C30G70-PDA groups were associated with fewer CD206+ cells and more iNOS+ cells, especially the C15G85 group. Quantitative analysis showed that the ratios of M1/M2 cells in the C15G85, C0G100-PDA, C15G85-PDA and C30G70-PDA groups were 8.08 ± 0.14, 3.41 ± 0.14, 0.38 ± 0.023 and 4.06 ± 0.05, respectively (Figure 5C). The findings suggested that the C15G85-PDA grafts exhibited a higher efficacy in inducing M2 macrophages polarization when compared to the C15G85, C0G100-PDA and C30G70-PDA groups. Collectively, the infiltration of macrophages around the C15G85-PDA graft created an early favorable immune microenvironment for bone regeneration. Combined with the stable degradation rate, the C15G85-PDA graft may offer a matched and long-lasting immunomodulatory platform for PDA interface to enable macrophages-ESCs crosstalk to complete the vertical full-thickness bone repair of critical-sized defects.

### scRNA-seq analysis identified genotypes of dural cells and macrophages

**Figure 6.**
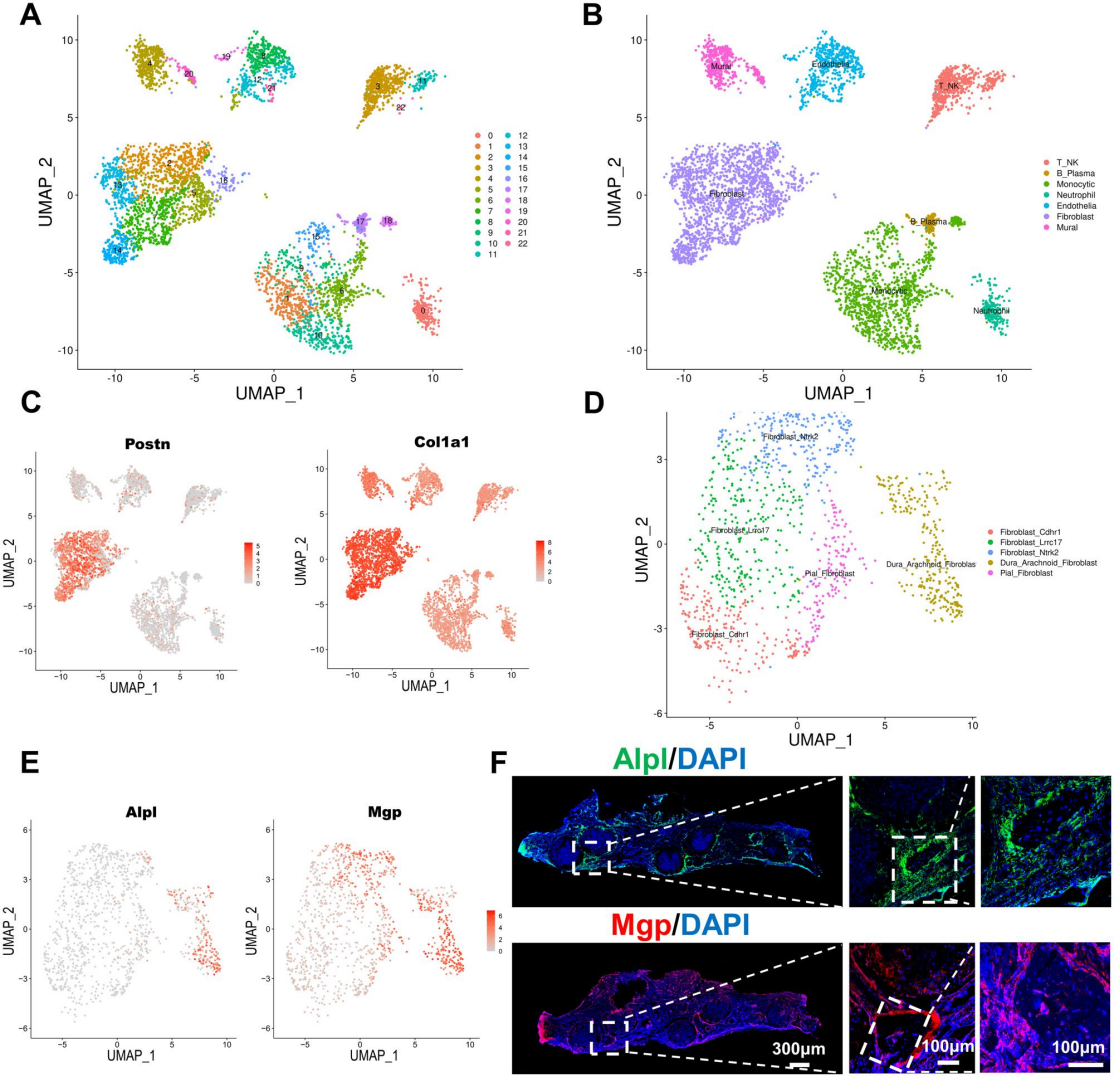
Single-cell RNA seq analysis of the C15G85-PDA group at 2 weeks. (**A**) UMAP analysis atlas of C15G85-PDA graft remodeling at 2 weeks after implantation. (**B**) Specific cluster identification map of the cell type. (**C**) Feature plots showed the expression of specific marker genes in fibroblast subsets. (**D**) Specific cluster identification map of Fibroblast. (**E**) Feature plots showed the expression of specific marker genes in Dura-Arachnoid Fibroblast subsets. (**F**) Representative immunofluorescent staining of Alpl and Mgp in regenerative tissue at 2 weeks.

To determine regulated cell subsets in response to the *in situ* culture system with C15G85-PDA grafts, scRNA-seq analysis of the *in situ* cell clusters was performed after implantation for 2 weeks. Cells of the regenerative tissues were isolated, and mRNA libraries were prepared (10× Genomics) and sequenced. Twenty-two subpopulations that occurred in the regenerative tissues were identified through integrated analysis (Figure 6A). All these subpopulations were genetically classified into endothelial (cluster 8, 12, 19 and 21, expressing Vwf and Cdh5), fibroblasts (clusters 2, 5, 7, 13, 14 and 16, expressing Col3a1 and Dcn), mural cells (clusters 4 and 20, expressing Acta2 and My19), neutrophils (cluster 0, expressing S100a9 and G0s2), B/plasma cells (cluster 17, expressing CD79a and Mzb1), monocytic cells (cluster 1, 6, 9, 10, 15 and 18, expressing C1qa) and T/NK cells(cluster 3, 11 and 22, expressing CD3d and CD3e) *via* uniform manifold approximation and projection (UMAP) analysis (Figure 6B and Supplementary Figure S14). Moreover, macrophages (expressing CSF1R and CD68) derived from monocytic, neutrophil and B/plasma cells were classified into Mrc-1-macrophage (expressing Mrc1 and C1qa; Mrc1 is CD206), IL1b-macrophage (expressing IL1b and Ptgs2), Spp1—macrophage (expressing Spp1 and Acp5), Ccl17—macrophage (expressing Ccl17 and Cfp), cycling macrophage (expressing Mki67 and Ube2c) and osteoclast (expressing Mmp9 and Ctsk) by sub-clustering analysis through clustering analysis again (Supplementary Figures S15 and S16).

In addition, Postn and Col1a1 were highly expressed in fibroblasts. Combined with the featured cell markers that were reported previously, fibroblasts could be defined as osteo-lineage cell population (expressing Col1a1 and Postn) (Figure 6C) [35]. To further study the characteristics of fibroblasts involved in bone remodeling, all fibroblasts were grouped into five types by sub-clustering analysis, they are: (1) Dura-Arachnoid fibroblasts (expressing Alpl, Fxyd5 and Mgp), its dural cells [36], (2) Pial fibroblasts (expressing Col18a1 and Spon1), its pial cells, (3) Cdhr1 fibroblasts (expressing Cdhr1 and CD55), (4) Lrrc17 fibroblasts (expressing Lrrc17 and Efemp1), (5) Ntrk2 fibroblasts (Ntrk2 and Apoe) (Figure 6D and E and Supplementary Figure S17). Immunofluorescence staining of Alpl and Mgp (markers of dural cells) in the newly formed tissues of C15G85-PDA grafts at 2 weeks showed that large numbers of dural cells migrated vertically from the meningeal side to the skin side (Figure 6F), which was consistent with the results of H&E staining at 4 weeks (Figure 4B). This further indicated that the main source of osteoblasts recruited by the C15G85-PDA graft was dural cells. Furthermore, as illustrated in Figures 5B and 6F, the regenerated tissue of the C15G85-PDA group at 2 weeks exhibited the existence of both dural cells and M2-dominant macrophages. The collective findings presented a comprehensive atlas of the regenerative-state of bone healing at a single-cell resolution, providing a thorough understanding of cellular heterogeneity within various cell types, especially focus on the osteo-lineage cells relevant to rat calvarial defect repair.

### C15G85-PDA elastomer recruited dural cells and enabled rapid calvarial bone regeneration

**Figure 7.**
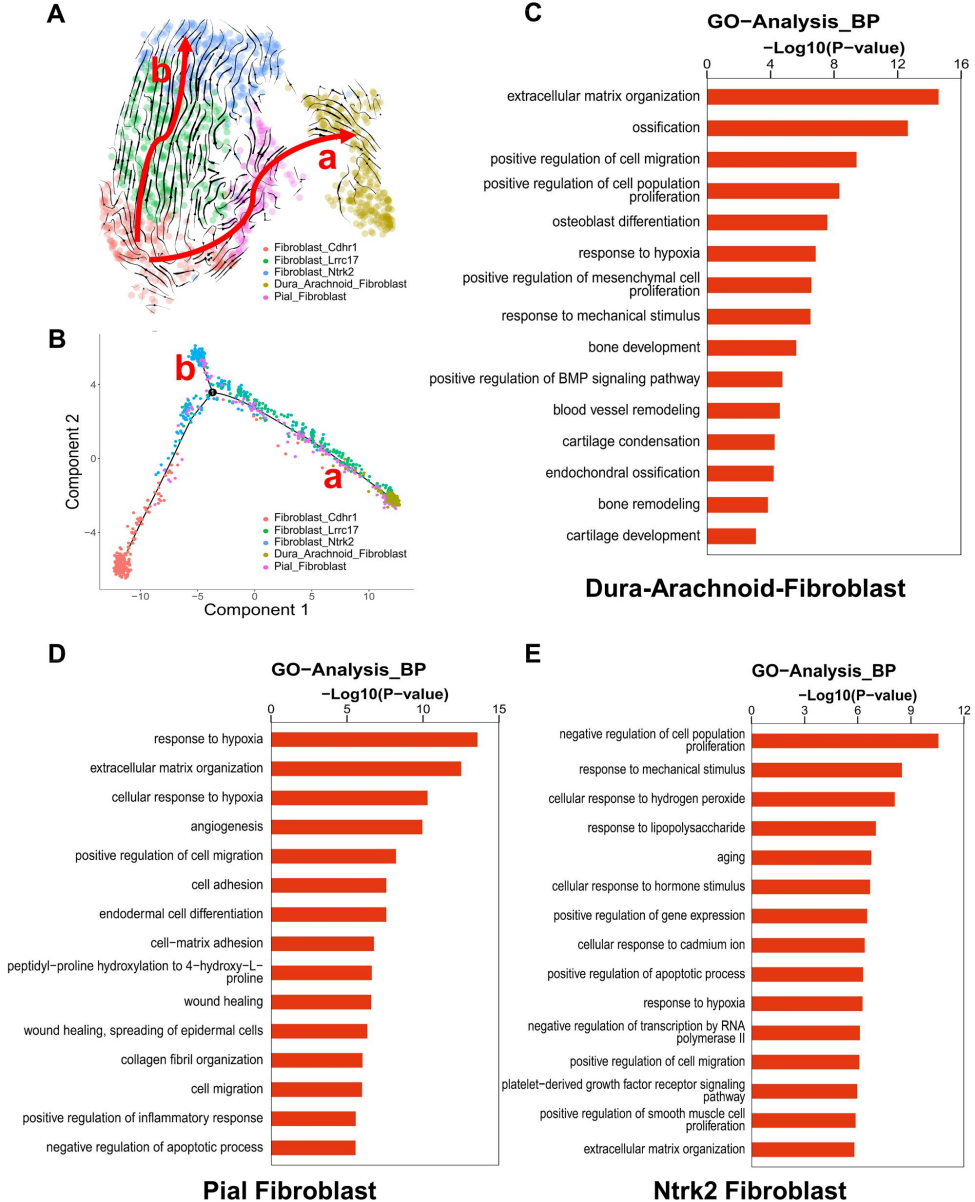
Single-cell RNA seq analysis of the C15G85-PDA group at 2 weeks. (**A**) Differentiation trajectory inferred by RNA velocity of the C15G85-PDA group after 2 weeks of defect surgery. Red arrow indicates the differentiation direction. (a) and (b) represent two distinct cell differentiation trajectories. (**B**) Trajectory of differentiation from Cdhr1-Fibroblasts to Dura-Arachnoid Fibroblasts (a) and Nkrk2-Fibroblasts (b) lineages predicted by Monocle. Barplot showed the GO enrichment in (**C**) Dura-Arachnoid Fibroblasts, (**D**) Pial Fibroblasts and (**E**) Nkrk2-Fibroblasts subset.

To further study the role of dural cells in bone regeneration and the lineage relationships among the osteo-lineage cell subsets, the pseudo-time analysis of fibroblasts was performed by RNA velocity. A significant branch point that altered the cell fate of Cdhr1 fibroblasts toward Dura-Arachnoid fibroblasts-downstream and Ntrk2 fibroblasts-downstream lineages was identified (Figure 7A). It is also revealed that Pial fibroblasts were located upstream of Dura-Arachnoid fibroblasts. To verify the findings, the pseudo-time analysis was also conducted using the Monocle 2 R package. The results showed that Cdhr1 fibroblasts were situated at the apex of the differentiation hierarchy, and there were strong directional streams from Cdhr1 fibroblasts toward Dura-Arachnoid fibroblasts and Ntrk2 fibroblasts, which formed two differentiation trajectories (Figure 7B). It was consistent with the RNA velocity analysis.

GO enrichment analysis was performed on the DEGs of the Dura-Arachnoid fibroblasts, Pial fibroblasts and Ntrk2 fibroblasts subset. The Dura-Arachnoid fibroblasts exhibited significant enrichment in processes such as extracellular matrix organization, ossification, positive regulation of cell migration, positive regulation of cell population, osteoblast differentiation, response to hypoxia, response to mechanical stimulus, bone development, blood vessel remodeling, cartilage condensation, endochondral ossification, bone remodeling and cartilage development (Figure 7C). Almost all biological processes were closely related to osteogenesis. In particular, ossification and endochondral ossification corresponded to the two osteogenesis patterns previously mentioned, intramembranous and endochondral ossification. The biological processes of Pial fibroblasts were response to hypoxia, extracellular matrix organization, angiogenesis, positive regulation of cell migration, cell adhesion, cell–matrix adhesion, wound healing, collagen fibril organization, cell migration and positive regulation of inflammatory response (Figure 7D), and the biological processes of Ntrk2 fibroblasts involved response to mechanical stimulus, response to hypoxia, positive regulation of cell migration and platelet-derived growth factor receptor signaling, suggesting that only part of their biological processes were indirectly related to osteogenesis, especially Ntrk2 fibroblasts (Figure 7E). Meanwhile, the Dura-Arachnoid fibroblasts exhibited a significant enrichment of Msx1, Msx2, Foxc1 and Foxc2, which are known to regulate craniofacial development and odontogenesis (Supplementary Figure S18A and B) [37–40]. Furthermore, Dura-Arachnoid fibroblasts and Pial fibroblasts also showed CD200 (a cell surface marker of mouse skeletal stem cells) and Runx2 (an early marker for osteogenesis, expressing in preosteoblasts and immature osteoblasts) enrichment (Supplementary Figure S18C) [41, 42]. Overall, the dural cells subset responded to the C15G85-PDA graft and was responsible for osteogenesis *in vivo*, which may be major osteoprogenitors of bone healing.

### Crosstalk between dural cells and macrophages played a vital role in bone healing

**Figure 8.**
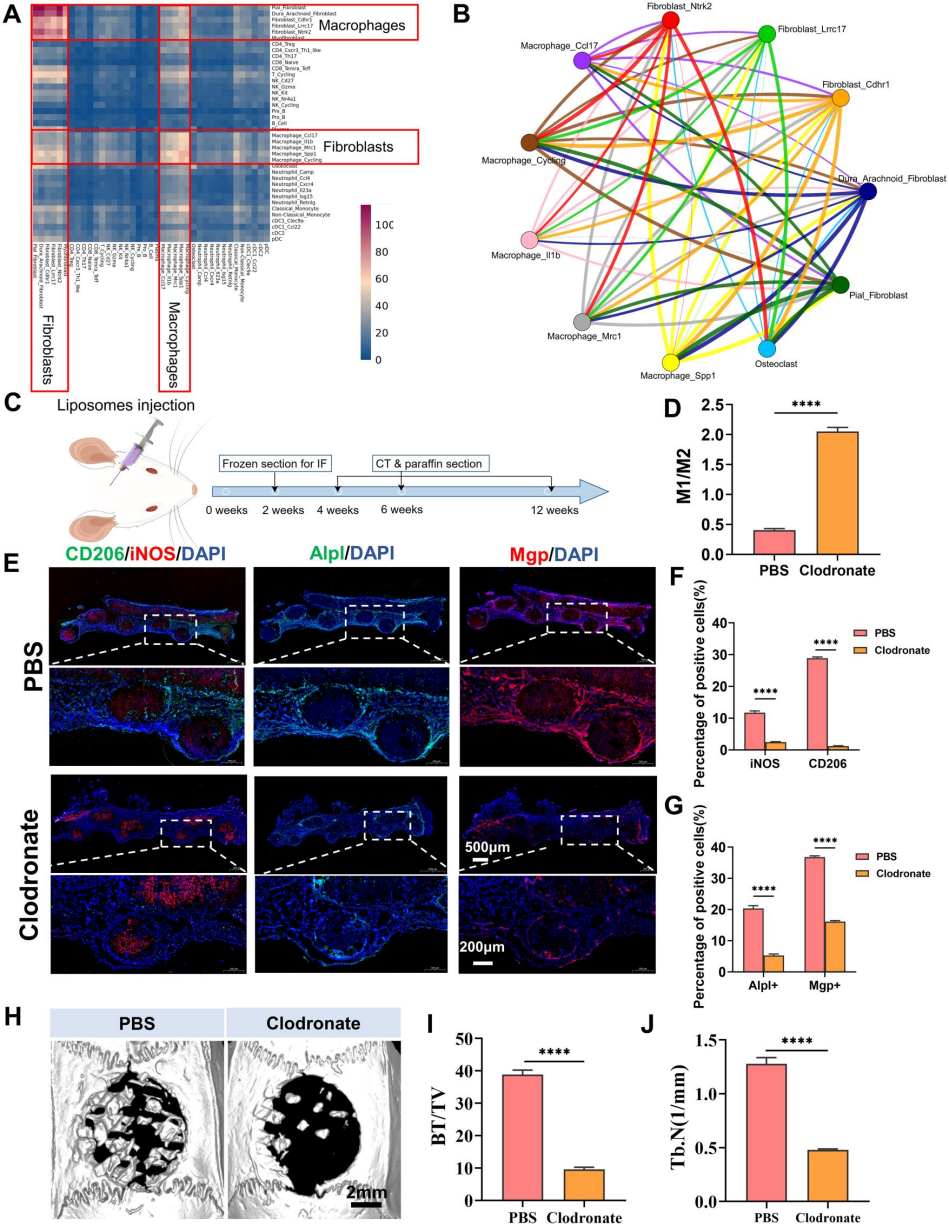
Macrophage depletion impaired the bone repair by C15G85-PDA graft. (**A**) Heatmap of cell–cell communication analysis based on CellPhoneDB. (**B**) Strength of ligand–receptor interactions among macrophages, Dura-Arachnoid Fibroblasts and Pial Fibroblasts pairs based on CellChat analysis. (**C**) Schematic diagram of the C15G85-PDA group loaded with PBS liposomes or clodronate liposomes into a rat calvarial defect. (**D**) Quantitative comparison of M1/M2 (*n* = 5). (**E**) Representative immunofluorescent of CD206, iNOS, Alpl and Mgp in regenerative tissue in the C15G85-PDA + PBS and C15G85-PDA + Clodronate at 2 weeks. (**F**) Quantitative comparison percentage of positive CD206 and iNOS cells (*n* = 5). (**G**) Quantitative comparison percentage of positive Alpl and Mgp cells (*n* = 5). (**H**) Micro-CT imaging analysis and (**I**) quantitative analysis of BV/TV and (**J**) Tb.N at 6 weeks in the C15G85-PDA + PBS and C15G85-PDA + Clodronate groups (*n* = 5). Edge width is proportional to the number of ligand–receptor pairs. Circle sizes are proportional to the number of cells per cluster. Data are presented as mean ± SD. ns, *P *>* *0.05; *P <* *0.05; **P <* *0.01; ***P <* *0.001; ****P <* *0.0001.

CellPhoneDB analysis of fibroblasts, macrophages, neutrophils, B/plasma cells, monocytic cells and T/NK showed that fibroblasts and macrophages had dominant communication hubs (Figure 8A). To investigate the role of macrophages in the process of bone regeneration, an analysis of macrophage receptor–ligand interaction was conducted utilizing the CellChat platform. The results of the CellChat analysis revealed that the recruited macrophages communicated frequently with dural cells (Figure 8B).

To further identify the critical role of the host macrophages played in the bone healing of the C15G85-PDA group, clodronate liposomes were used as a scavenger of macrophages in the surgical area of rat calvarial defects (Figure 8C). Frozen sections were used for immunofluorescence staining, and the results showed that all quantitative results of M1 and M2 macrophages were decreased dramatically in the C15G85-PDA + Clodronate group, compared to the C15G85-PDA + PBS group (Figure 8D–F). Meanwhile, Alpl+ and Mgp+ cells reduced significantly in the C15G85-PDA + Clodronate group, suggesting the relationship between dural cells and macrophages was ‘live together and die together’ (Figure 8G). It was confirmed that the depleting macrophages affected the recruitment of dural cells, especially M2 macrophages and their crosstalk was critical in the process of bone repair. At 4, 6 and 12 weeks after implantation and injection, compared with the new bone in the C15G85-PDA + PBS group, the new bone diminished significantly in the C15G85-PDA + Clodronate group (Figure 8H–G and Supplementary Figure S19A–C). H&E staining and Masson’s Trichrome staining confirmed that depleting macrophages delayed bone regeneration significantly (Supplementary Figures S19 and S20D and E). Taken together, the crosstalk between dural cells and macrophages played an indispensable role in the bone healing of C15G85-PDA elastomer, especially M2 macrophages.

## Discussion

Currently, the successful healing of critical-sized calvarial bone defects remains a considerable challenge [11, 35]. Critical-sized calvarial bone defects are defined as bone defects where spontaneous regeneration is not expected without intervention. Typical critical calvarial bone defects of rats include two sizes: 5 and 8 mm [12, 35, 43, 44]. The 8 mm defect was chosen for this study because it spanned across the middle cranial suture and excluded the suture mesenchymal cells that played a regenerative role in the bone repair process in this area [45], thus making it more challenging. It has been proven that ‘M2 macrophages predominant’ response is favorable for the bone regeneration facilitated by biomaterials. Various biological immune materials, which aim to regulate the immune microenvironment for osteoprogenitors, have been researched [12–16, 43, 44]. However, most of the studies focused on modifying materials to regulate immune microenvironment, with limited research on the substrate materials themselves, which could impact the recruitment of ESCs by the form, porosity, filling rate and degradation rate of graft. Therefore, a series of PCL/PGS substrate materials were designed for PDA interface, as filling materials, to optimize the substrate material with suitable microstructures, opening channels and matched degradation rate that can effectively modulate macrophage type and fully recruit ESCs to achieve more complete repair of critical-sized defects.

3D printing has been widely adopted for producing grafts with customizable shapes and internal microstructures to individually meet the clinical needs. It could finely control the ratio of PGS and PCL, resulting in the tunable porosity and channels [46]. The microporous structure and channels not only provided space for cell infiltration but also offered abundant binding sites for PDA. In this study, the filling rate of all grafts was 25% to ensure sufficient opening channels between the columns, thereby enabling the infiltration of significant amounts of early immune cells and dural cells into the grafts. The porosity and the number of binding sites for PDA decreased with the increased PCL content, while the mechanical properties of grafts enhanced with the increased PCL content. Furthermore, the graft with more PDA nanoparticles had better hydrophilicity, which favored cell adhesion and proliferation [47, 48]. Given the above results, it was rational to deduce that the C15G85-PDA graft may have excellent osteogenic efficiency.

3D printing approach not only resulted in the tunable porosity and channels, but also the tunable degradation rate. Previous studies revealed that PGS almost degraded within 2 weeks, while some PCL residue remained until 12 weeks [49, 50]. It was also reported that either too rapid or too slow degradation rate of grafts would result in suboptimal bone repair [51–55]. The matched degradation rate is therefore the prerequisite for bone regeneration. As shown in Figure 2A, the degradation rate of graft was positively correlated with the degree of cell infiltration. The C15G85-PDA graft exhibited a lower degradation rate and a lower degree of cell infiltration compared to those of the C30G70-PDA graft. There are two possible reasons for this phenomenon: (i) The weaker mechanical properties of the C15G85-PDA group, as shown in Figure 1H and I, led to the compression of micropores in the graft due to surrounding tissue pressure, hindering the infiltration of cell around the graft. (ii) It was found that the PGS/PCL graft closely resembled a ‘concrete’ construct. PCL can be likened to ‘sand’, while PGS can be likened to ‘cement’ in ‘concrete’. Due to less PGS in the C30G70-PDA group, the entire graft was more likely to disintegrate, allowing cells to infiltrate quickly compared to the C15G85-PDA group. Hence, the C15G85-PDA graft exhibited a lower degradation rate. The PDA interface of the C15G85-PDA graft was the most stable and matched, and the osteogenic efficiency was the best. It is a special degradation pattern in PGS/PCL construct for PDA interface. Although the degrading rate of grafts in the subcutaneous pouches was different from that implanted in the calvarial defects, the general trend was same (Figures 2A and 4A–C, Supplementary Figures S7A and S10A). The discrepancy of degrading behavior presented in grafts harvested from subcutaneous pouches and calvarial bone defects may be caused by variant anatomical locations and microenvironments [56, 57]. Moreover, the current study demonstrated that the degradation and phagocytosis of biodegradable materials after implantation were primarily facilitated by M1 macrophages [58, 59]. PDA coating delayed the overall degradation rate of the grafts in this study, which may be due to the predominance of M2 macrophages mediated by PDA. The delayed degradation rate was observed to be more matched with the rate of new bone formation. Together, 3D elastomers of the C15G85-PDA group enabled the matched degradation rate for PDA interface in parallel with dural cells infiltration in the bone healing process.

According to our previous results, the PDA-modified GBR membrane could not repair 8 mm calvarial bone defects effectively, which may be due to its non-contact with the dura mater and inability to effectively recruit osteoprogenitors from the dura mater (Supplementary Figure S1). The C15G85-PDA graft, as filling material, achieved full-thickness regeneration of 8 mm calvarial bone defect by fully recruiting dural cells. The vital contribution of dural cells was confirmed from three aspects: (i) A wide distribution of Alpl+ and Mgp+ dural cells in grafts of whole defects at 2 weeks. (ii) At 4 and 6 weeks, new bone initially formed was near the dura mater and gradually showed a vertical ‘C’-shaped ring regeneration pattern from the bottom to the top of the defects. (iii) The results of scRNA-seq directly confirmed that dural cells were closely related to bone regeneration. To the best of our knowledge, affirming the decisive contribution of PDA in recruitment of dural cells is new to bone substitutes. This report is the first step to identifying the role of the C15G85-PDA graft in recruiting dural cells in bone defects for calvarial bone regeneration.

Craniofacial bones originate from the cranial neural crest, and exhibit distinct characteristics from long bones in terms of their developmental origins, osteogenic processes and anatomical structures. They primarily develop through intramembranous osteogenesis rather than endochondral ossification, possess minimal marrow content and are enveloped by both periosteum and dura mater. Most studies involving calvarial regeneration aimed to mobilize BMSCs to form new bone through intramembranous ossification [60]. It was shown that the cranial suture stem cells characterized as Msx1+ cells assisted bone regeneration and might potentially differentiate into chondrocytes [35]. In this study, the rapid regeneration from dural cells may be due to the simultaneous endochondral and intramembranous ossification. Combined with histologic results, it was inferred that different microenvironments (inside the columns and between the columns) may determine the pattern of bone formation. It was known that intramembranous ossification took place at the periosteum, which benefited from a robust blood supply in proximity to the fracture site. Conversely, regions experiencing hypoxic conditions undergo endochondral ossification [61]. Therefore, variant oxygen concentrations in different part of the 3D elastomers, such as inside and outside the columns, might play a vital role in directing different osteogenesis patterns. When the inflammation subsided, the material in close contact with the dura mater fully recruited osteoprogenitors from the dura mater to differentiate into osteoblasts, producing organic osteoid matrix and initiating bone mineralization, while cartilage was formed inside the columns due to hypoxia, and gradually ossified with the degradation of the grafts and the formation of blood vessels.

When 3D elastomers were implanted into bone defects, they triggered immune responses that led to changes in the immune microenvironment necessary for bone regeneration. Various types of immune cells, including neutrophils, lymphocytes, dendritic cells and macrophages, actively contributed to the establishment of bone-immune microenvironment. Neutrophils were observed in the vicinity of implanted biomaterials during the early inflammatory phase, while macrophages assumed a predominant role in the interactions between the host and the graft [62, 63]. Prior studies have revealed that the involvement of M1/M2 macrophage subsets in the tissue healing procedures, and highlighted the relationship between macrophages and BMSCs for bone regeneration [64, 65]. The study confirmed that the C15G85-PDA grafts favorably guided M2 macrophages polarization around the PDA interface, while the other groups showed the M1 macrophages predominance. Additionally, both macrophages, Alpl+ and Mgp+ dural cells were presented in the regenerative tissue of the C15G85-PDA graft at 2 weeks, and further crosstalk between dural cells and macrophages was confirmed by CellPhoneDB analysis, while macrophage depletion by clodronate liposome resulted in the significant reduction of macrophages, Alpl+ and Mgp+ dural cells, which resulted in little bone healing. This is the first study to identify the relationship between dural cells and macrophages, thus offering the new target for further calvarial bone regeneration.

scRNA-seq allowed large-scale profiling of cell properties in complex tissues, revealing cellular heterogeneity in rat calvarial bone repair. Here, Alpl+ and Mgp+ dural cells were first identified in regenerative tissues of a rat calvarial defect at a single-cell level, which showed many biological processes related to osteogenesis and a distinct differentiation trajectory during injury repair by the pseudo-time analysis. The Alpl+ and Mgp+ dural cells subset also exhibited strong expression of various genes involved in craniofacial development in the regenerative tissue, such as Msx1, Msx2, Foxc1 and Foxc2. Recent studies have shown that Msx1 and Msx2 are strongly expressed in the developing craniofacial regions, while Foxc1 and Foxc2 genes are involved in the complex regulatory network of tooth and craniomaxillofacial bone development. Furthermore, Msx1, Msx2, Foxc1 and Foxc2 genes were highly expressed in the dura mater beneath the skull and in the suture mesenchyme during normal development [37–40]. These findings again suggested that osteoblasts mainly originated from the dura mater. Additionally, the findings indicated that the augmented regeneration of the calvarial bone involved a process of endochondral ossification, which exhibited a strong association with dural cells at a single-cell level, even though the calvarial bone is a well-known site of intramembranous ossification.

Although the C15G85-PDA graft effectively recruited dural cells to mimic the physiological calvaria formation process for bone regeneration, detailed mechanisms regulating crosstalk between dural cells and macrophages remain unclear and are worthy of further investigation.

## Conclusion

In summary, the study indicated that a matched and effective PDA interface was formed on a well-proportioned elastomer, which effectively modulated the polarization of M2 macrophages to promote the recruitment of dural cells to achieve full-thickness bone repair. The implementation of this study clarified the role of dural cells in the bone healing process and provided a single-cell level theory for the rapid regeneration of critical-sized bone defects. Moreover, owing to the 4D-morphing and instantly fixable capabilities of 3D elastomer, the C15G85-PDA graft offered a significant clinical alternative for cranial regeneration.

## Supplementary Material

rbae059_Supplementary_Data

## Data Availability

The data used to support the findings of this study are available from the corresponding authors upon request.
