# Supplementary figures and images for: Mussel inspired 3D elastomer enabled rapid calvarial bone regeneration through recruiting more osteoprogenitors from the dura mater
Source: Regen Biomater. 2024 May 22;11:rbae059. doi: 10.1093/rb/rbae059 (PMC11193312; doi:10.1093/rb/rbae059)

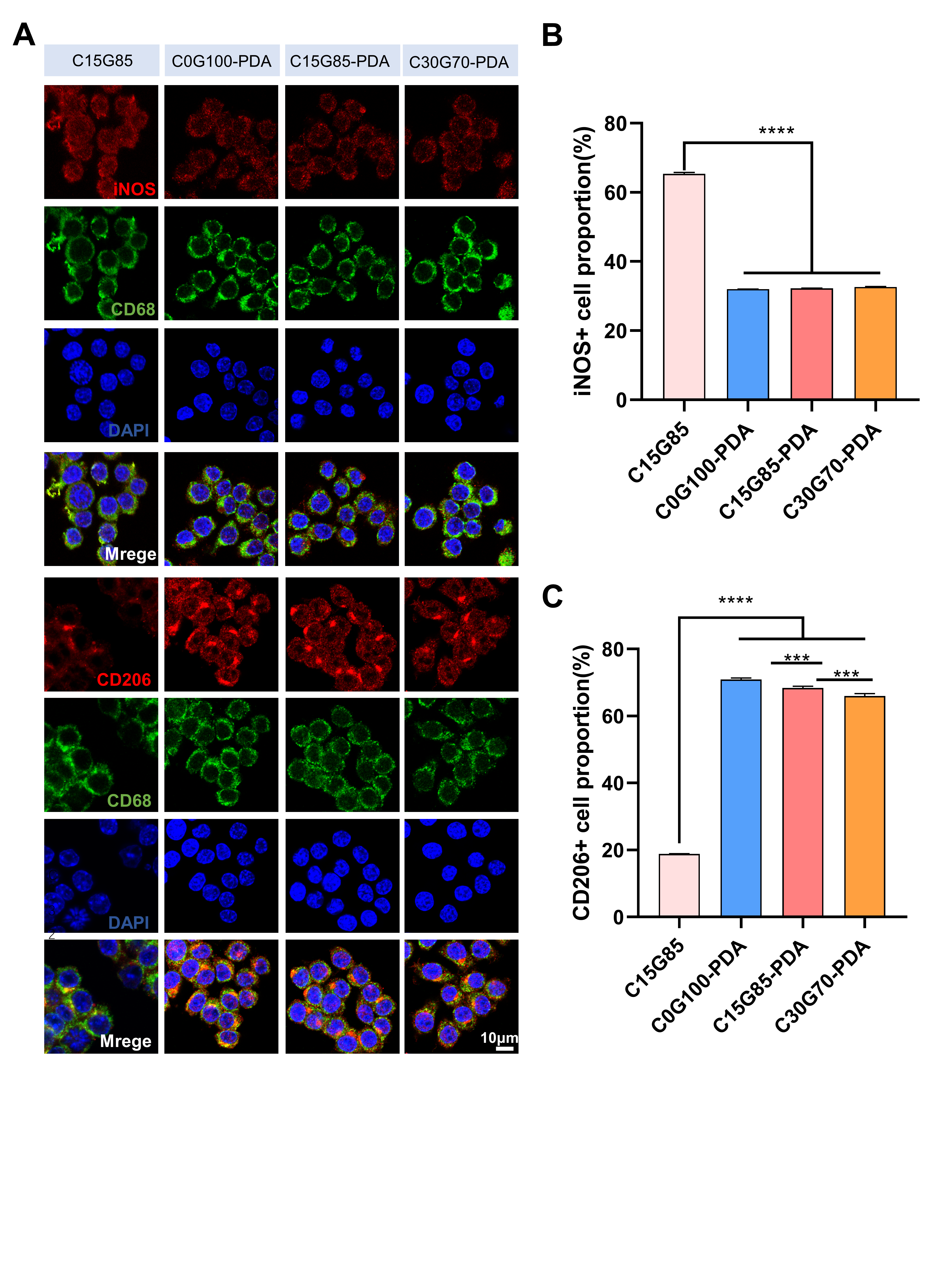

Supplement: rbae059_Supplementary_Data [file rbae059_supplementary_data.zip › Figure S13.tif]

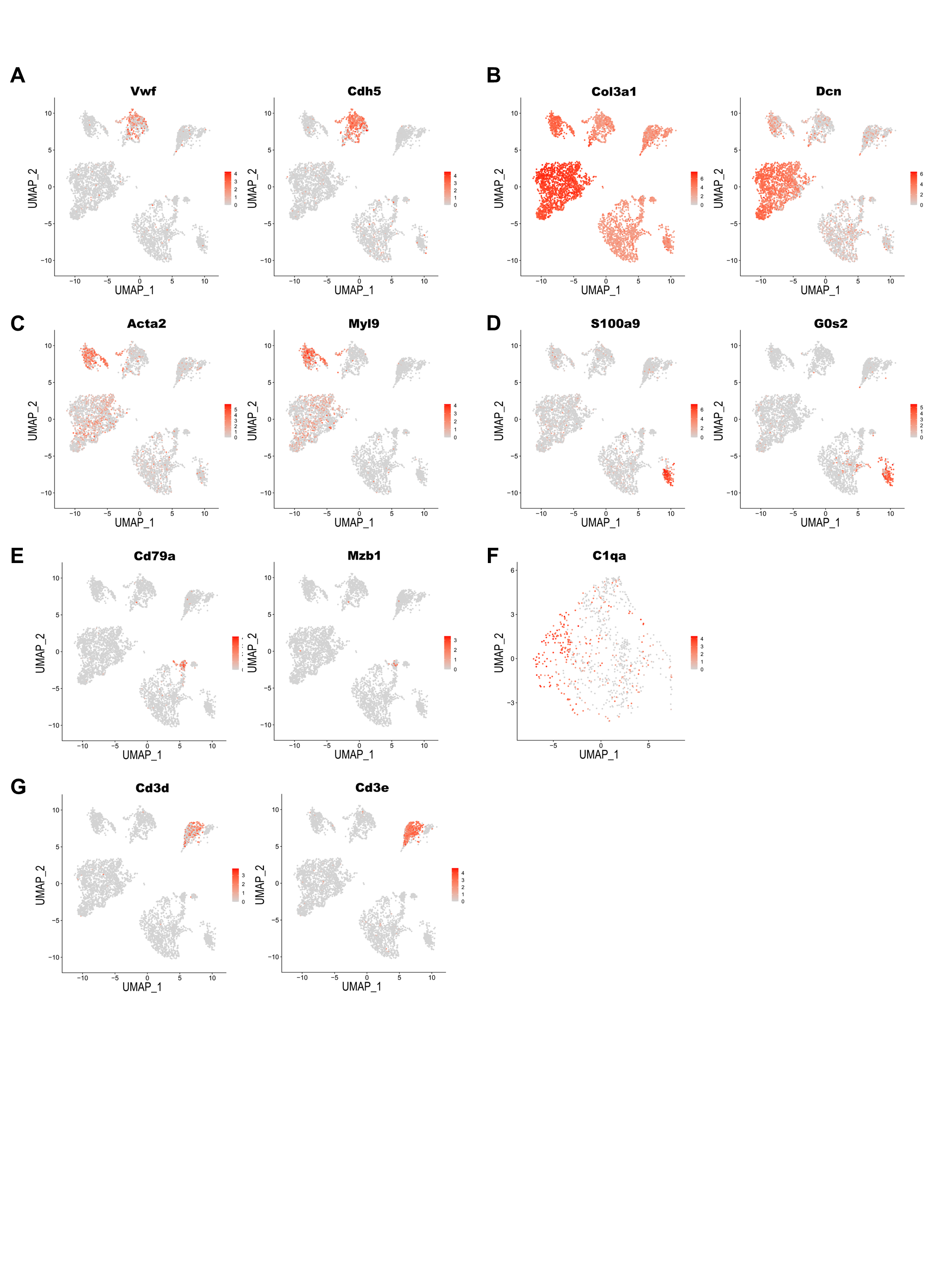

Supplement: rbae059_Supplementary_Data [file rbae059_supplementary_data.zip › Figure S14.TIF]

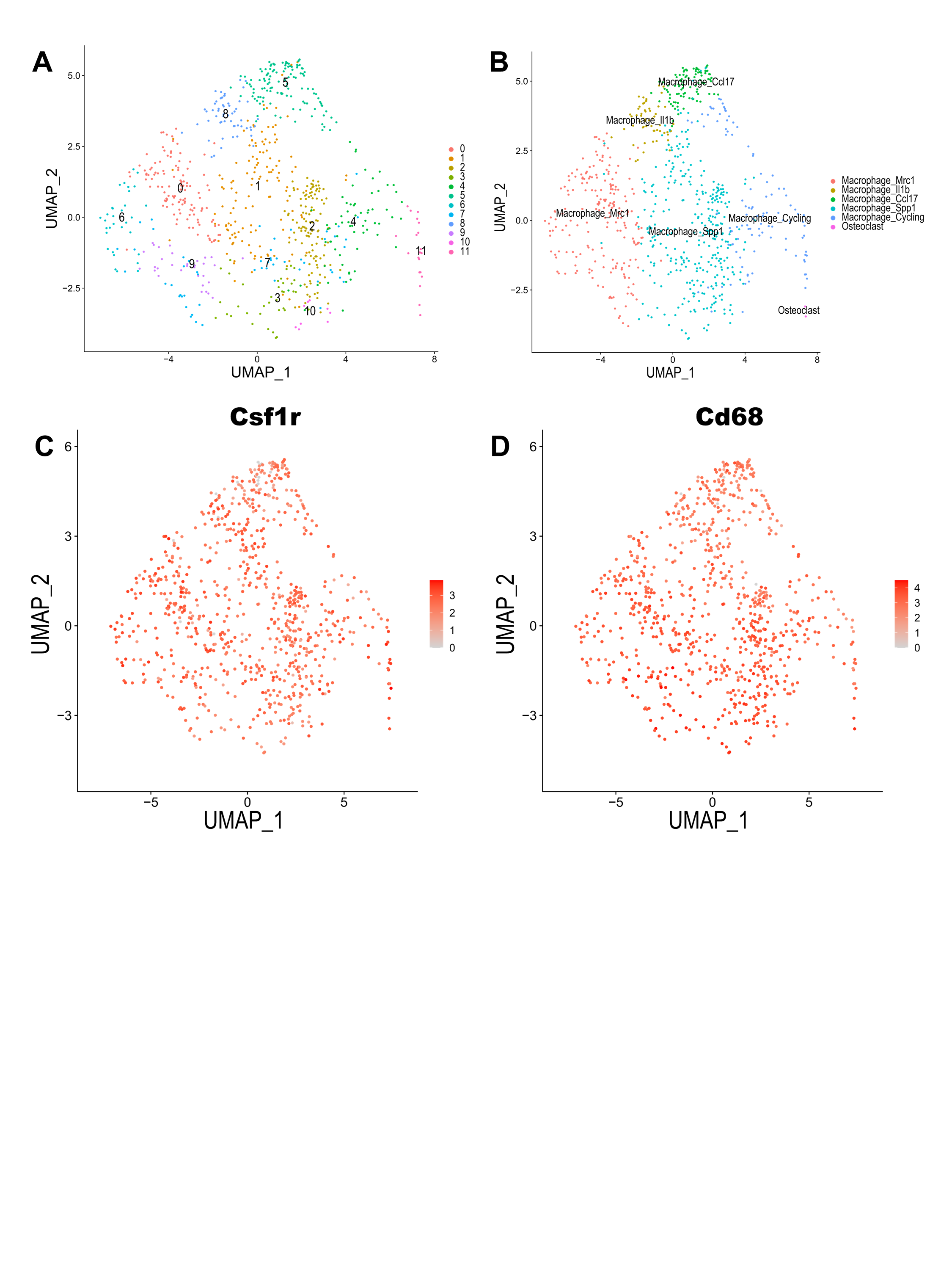

Supplement: rbae059_Supplementary_Data [file rbae059_supplementary_data.zip › Figure S15.TIF]

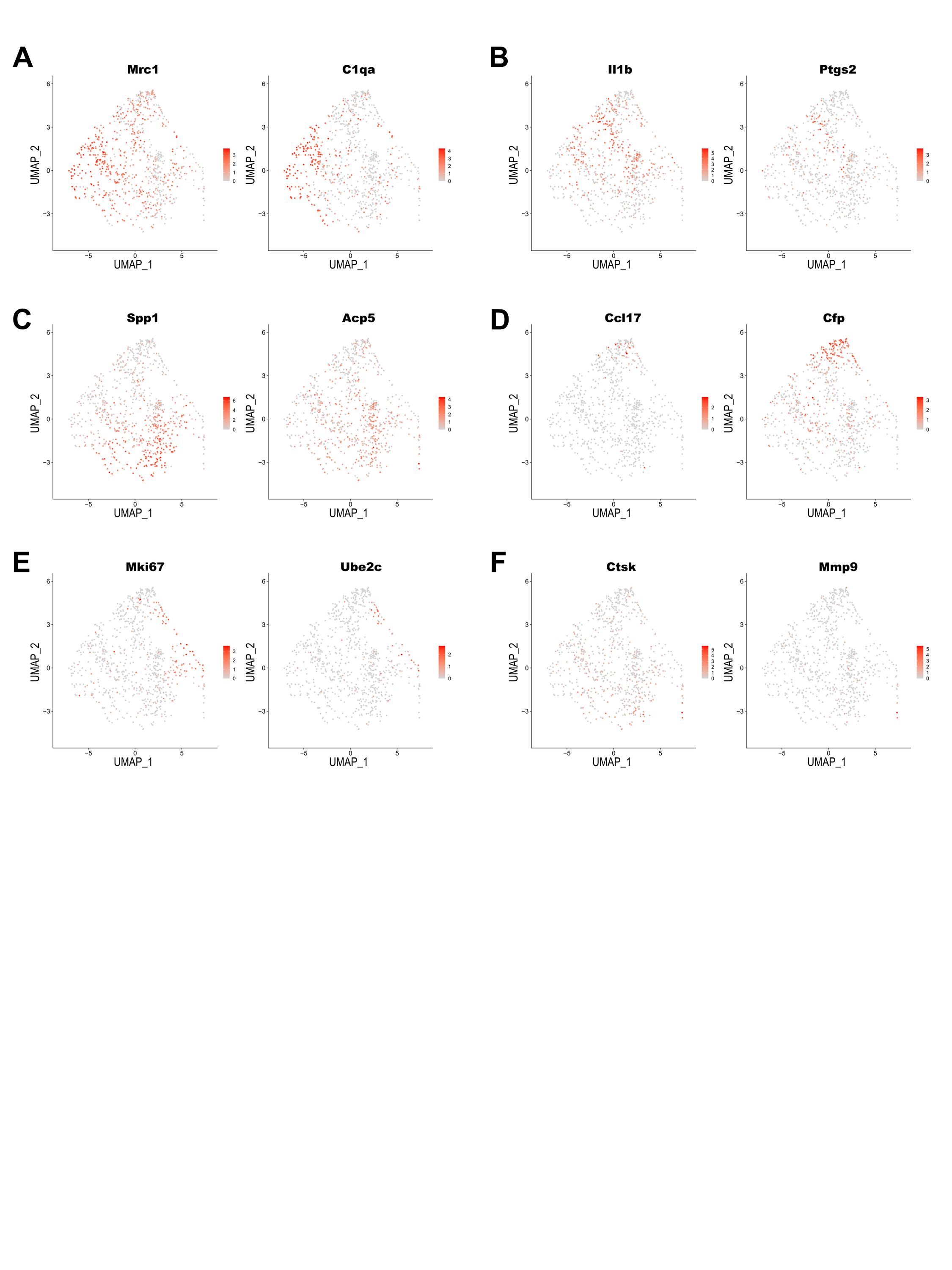

Supplement: rbae059_Supplementary_Data [file rbae059_supplementary_data.zip › Figure S16.TIF]

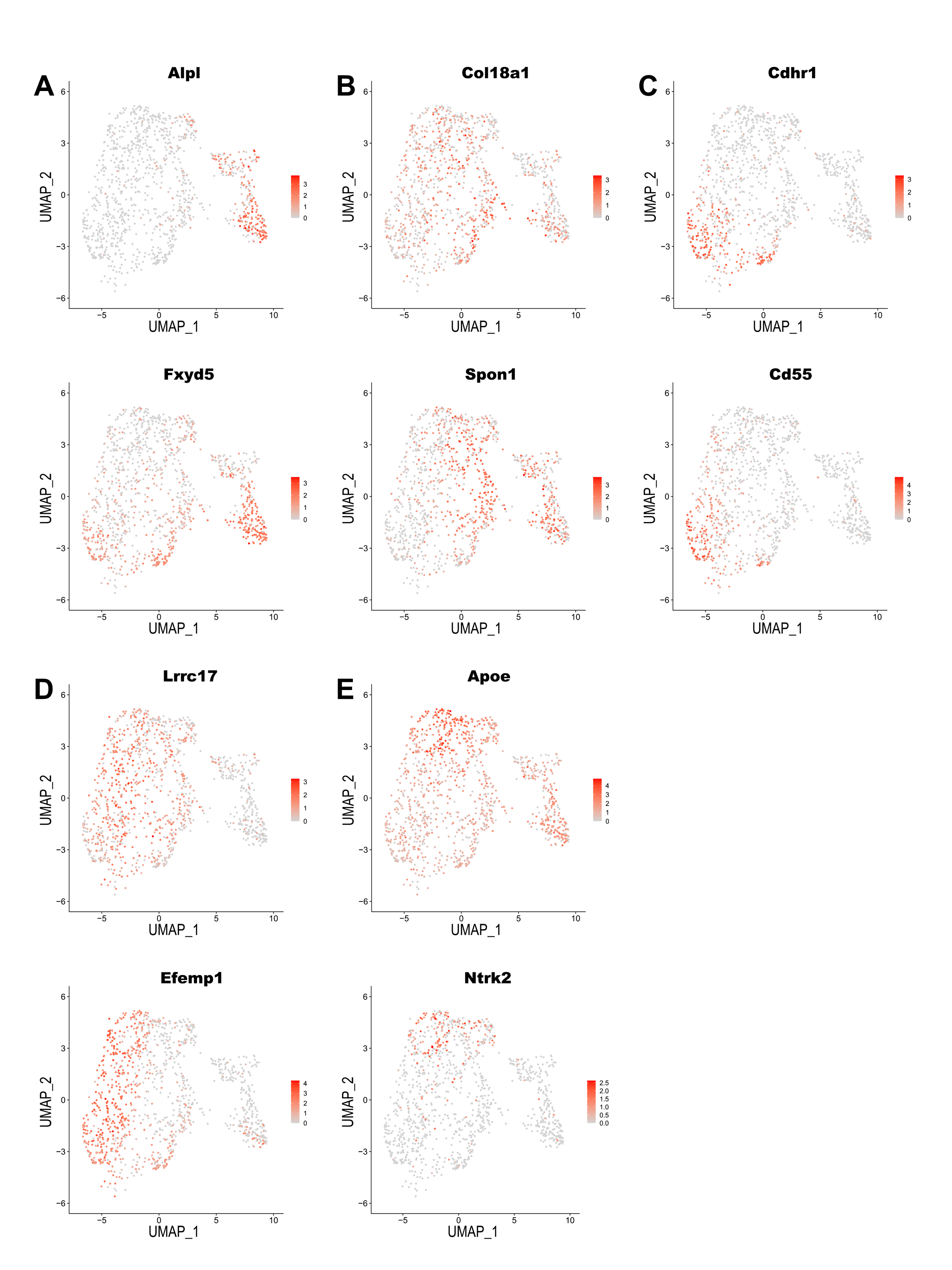

Supplement: rbae059_Supplementary_Data [file rbae059_supplementary_data.zip › Figure S17.TIF]

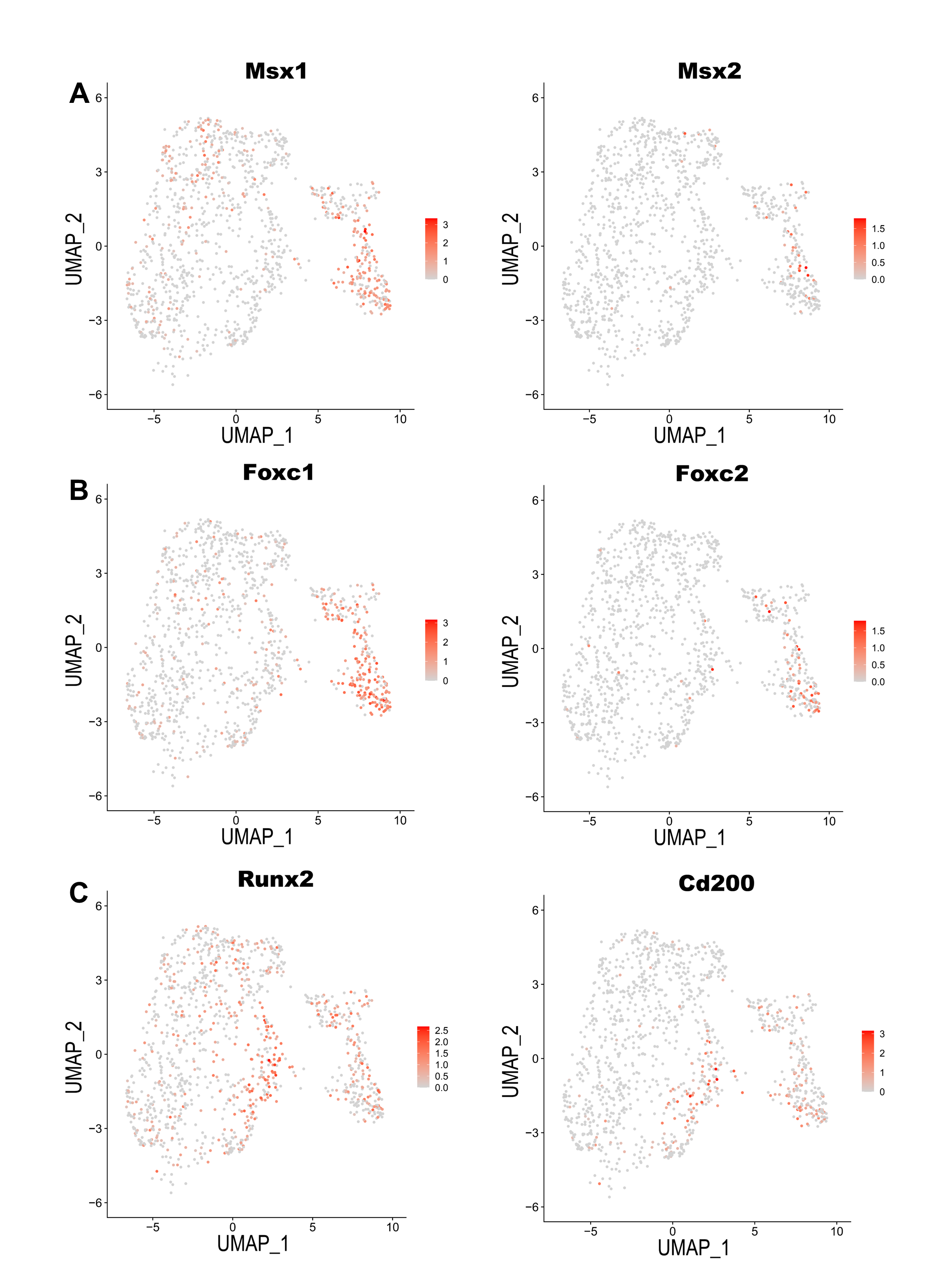

Supplement: rbae059_Supplementary_Data [file rbae059_supplementary_data.zip › Figure S18.TIF]

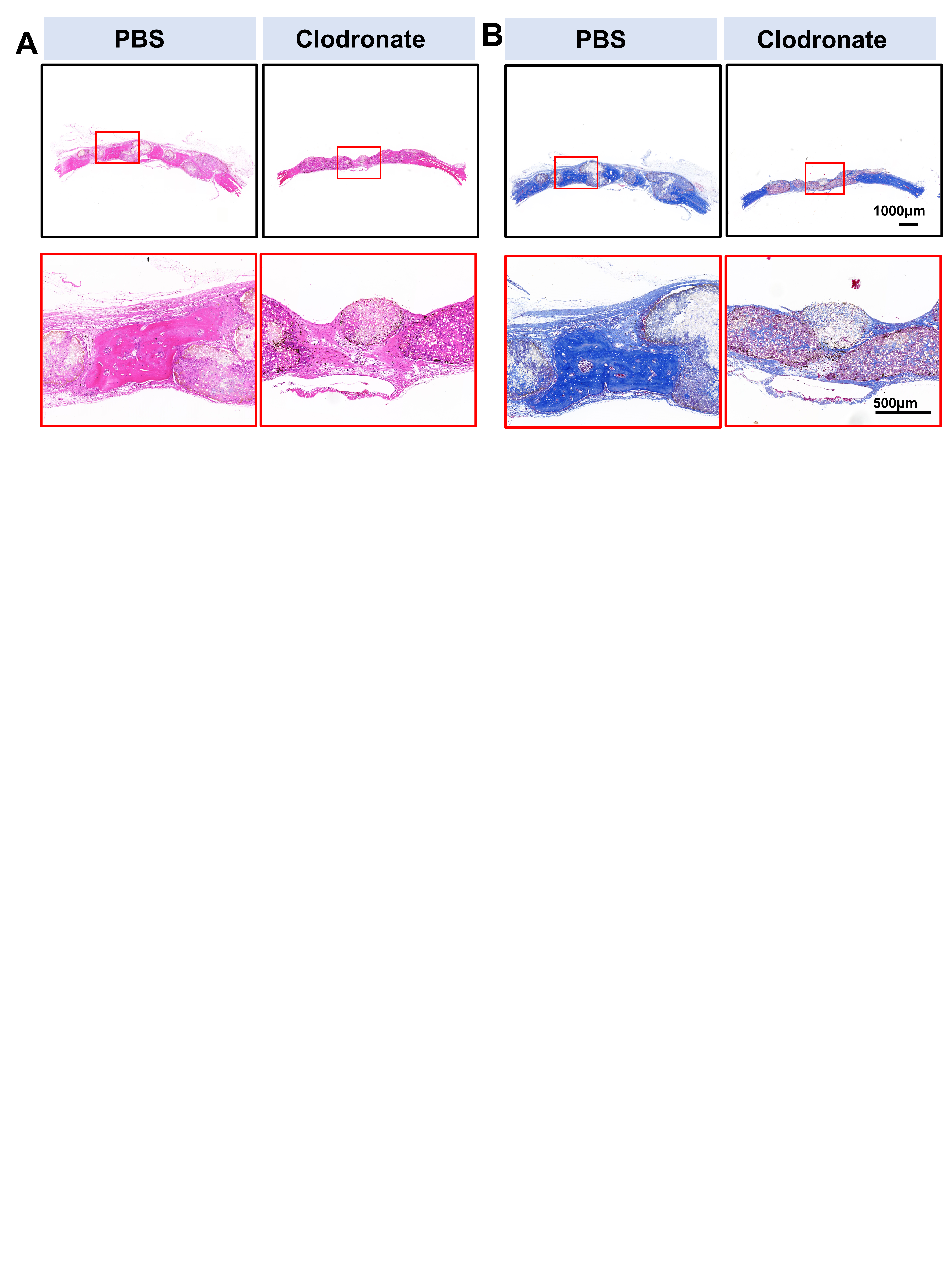

Supplement: rbae059_Supplementary_Data [file rbae059_supplementary_data.zip › Figure S19.tif]

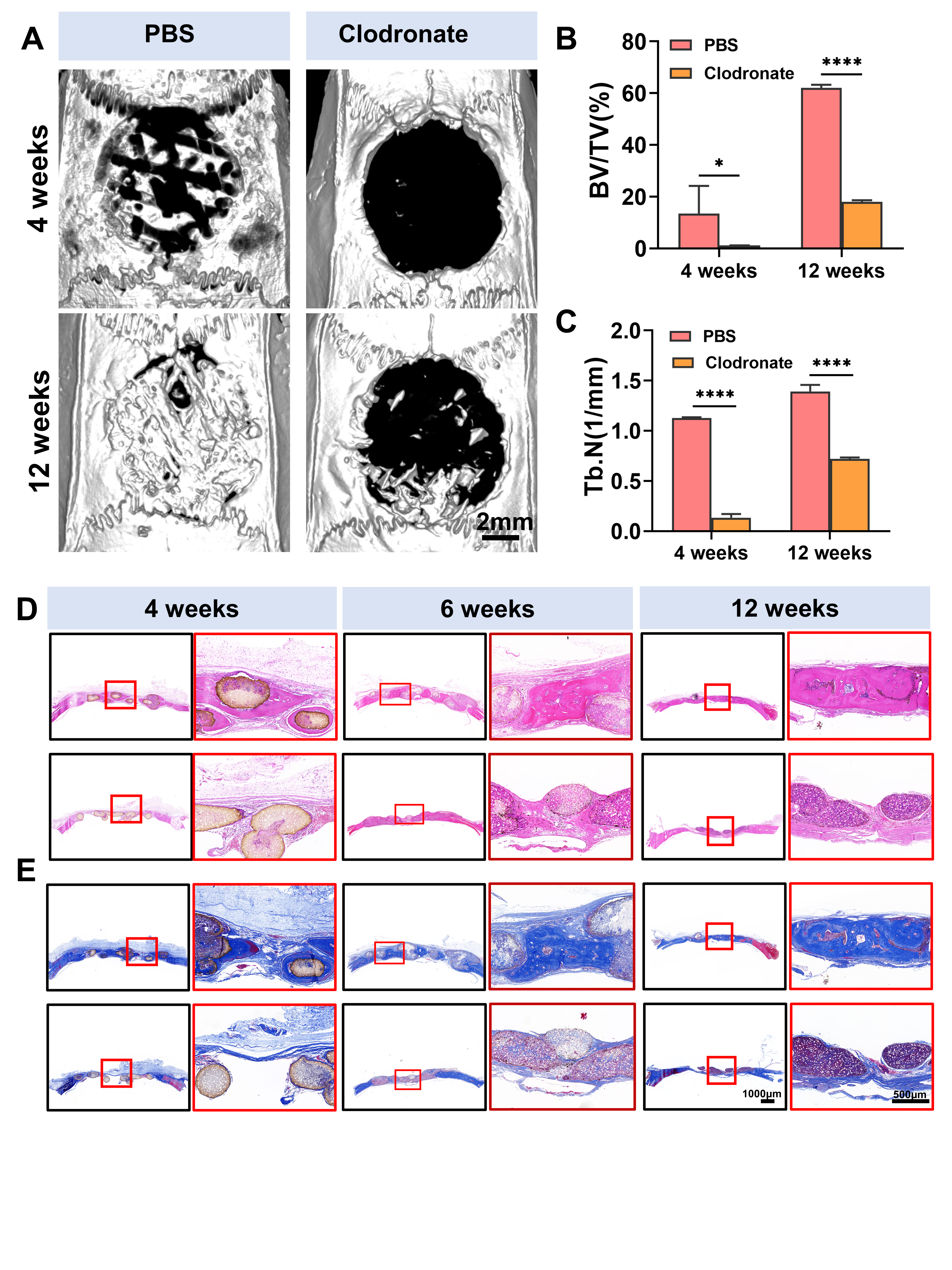

Supplement: rbae059_Supplementary_Data [file rbae059_supplementary_data.zip › Figure S20.tif]

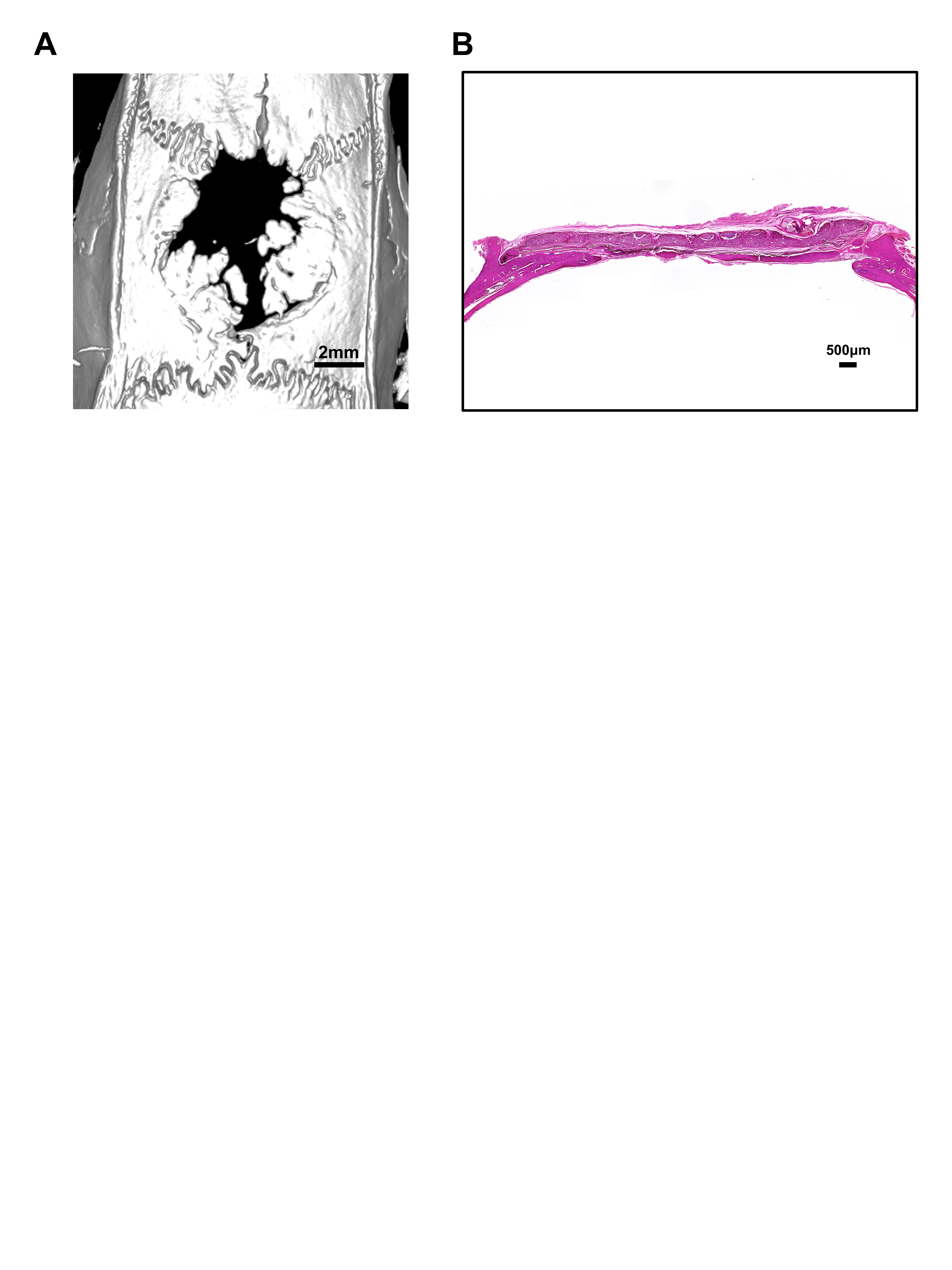

Supplement: rbae059_Supplementary_Data [file rbae059_supplementary_data.zip › Figure S1.tif]

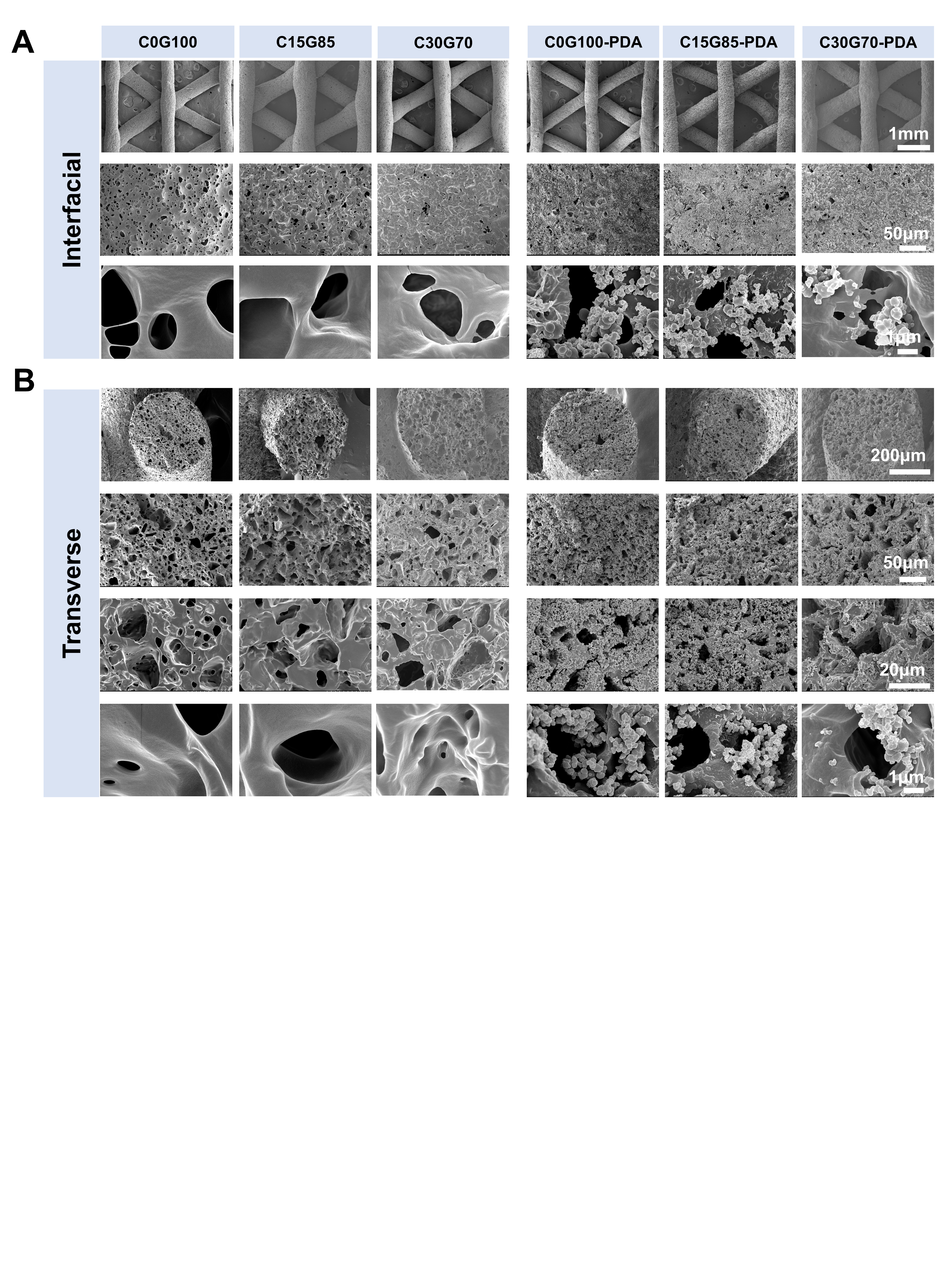

Supplement: rbae059_Supplementary_Data [file rbae059_supplementary_data.zip › Figure S2.tif]

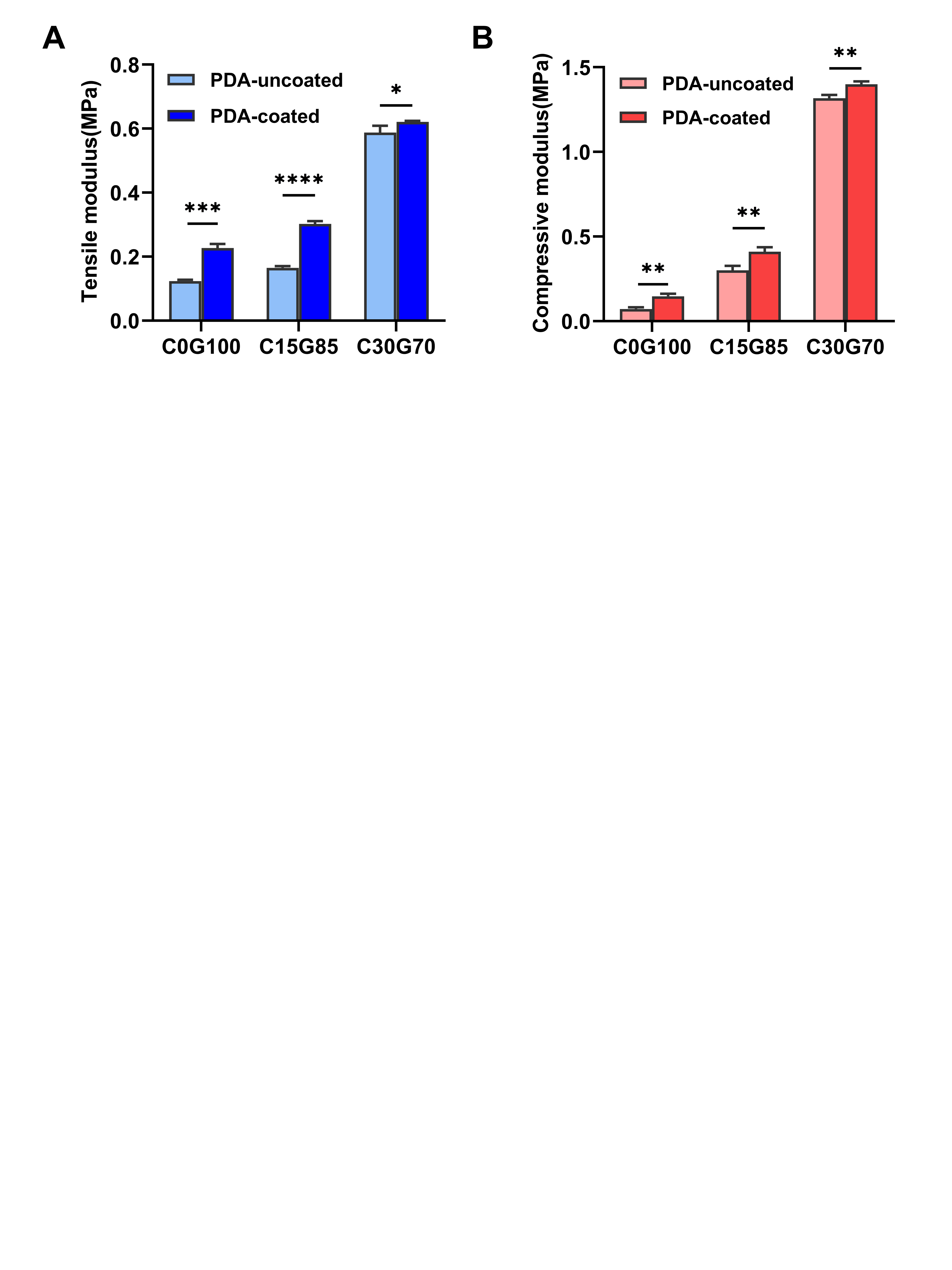

Supplement: rbae059_Supplementary_Data [file rbae059_supplementary_data.zip › Figure S3.tif]

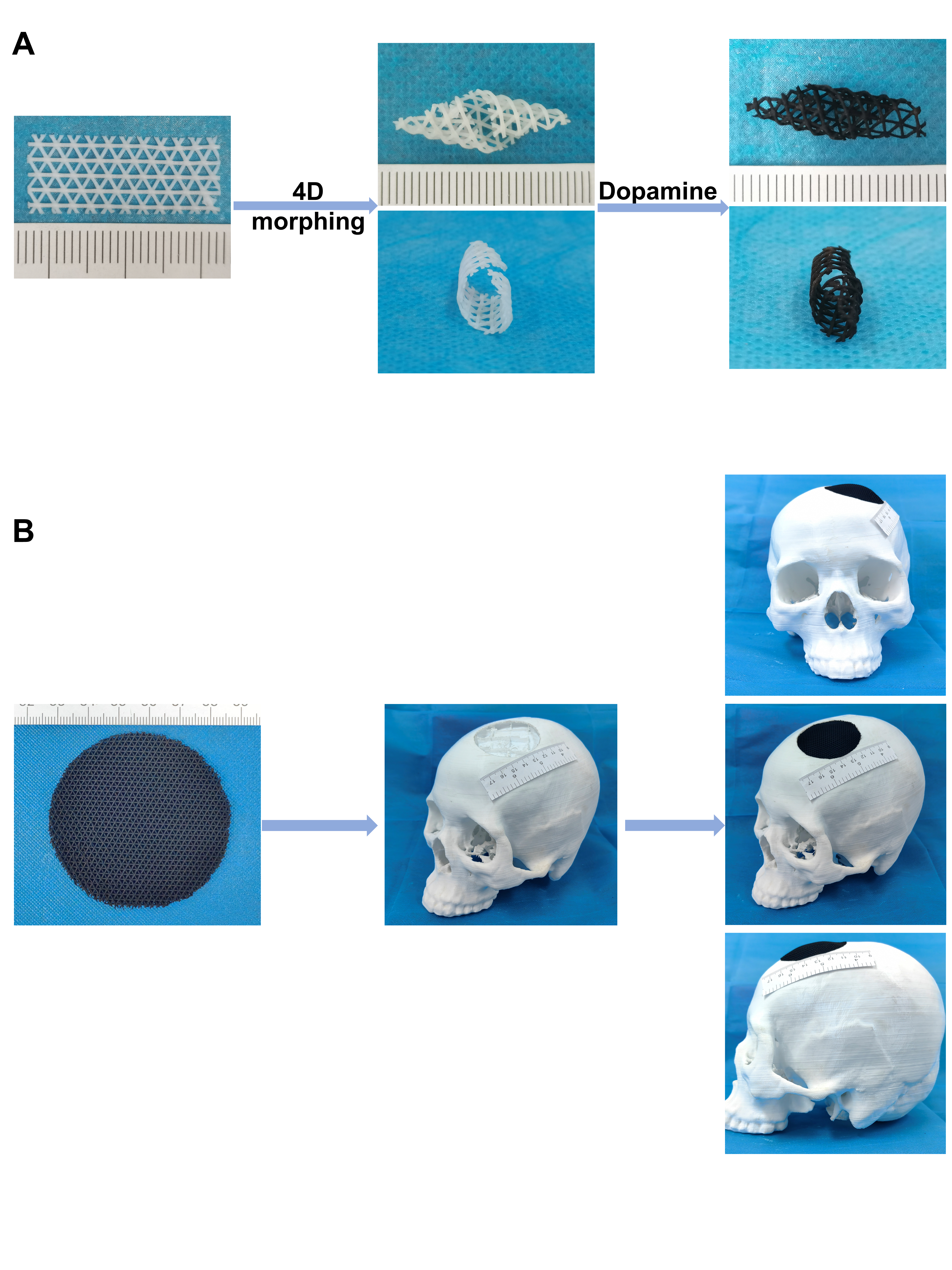

Supplement: rbae059_Supplementary_Data [file rbae059_supplementary_data.zip › Figure S4.tif]

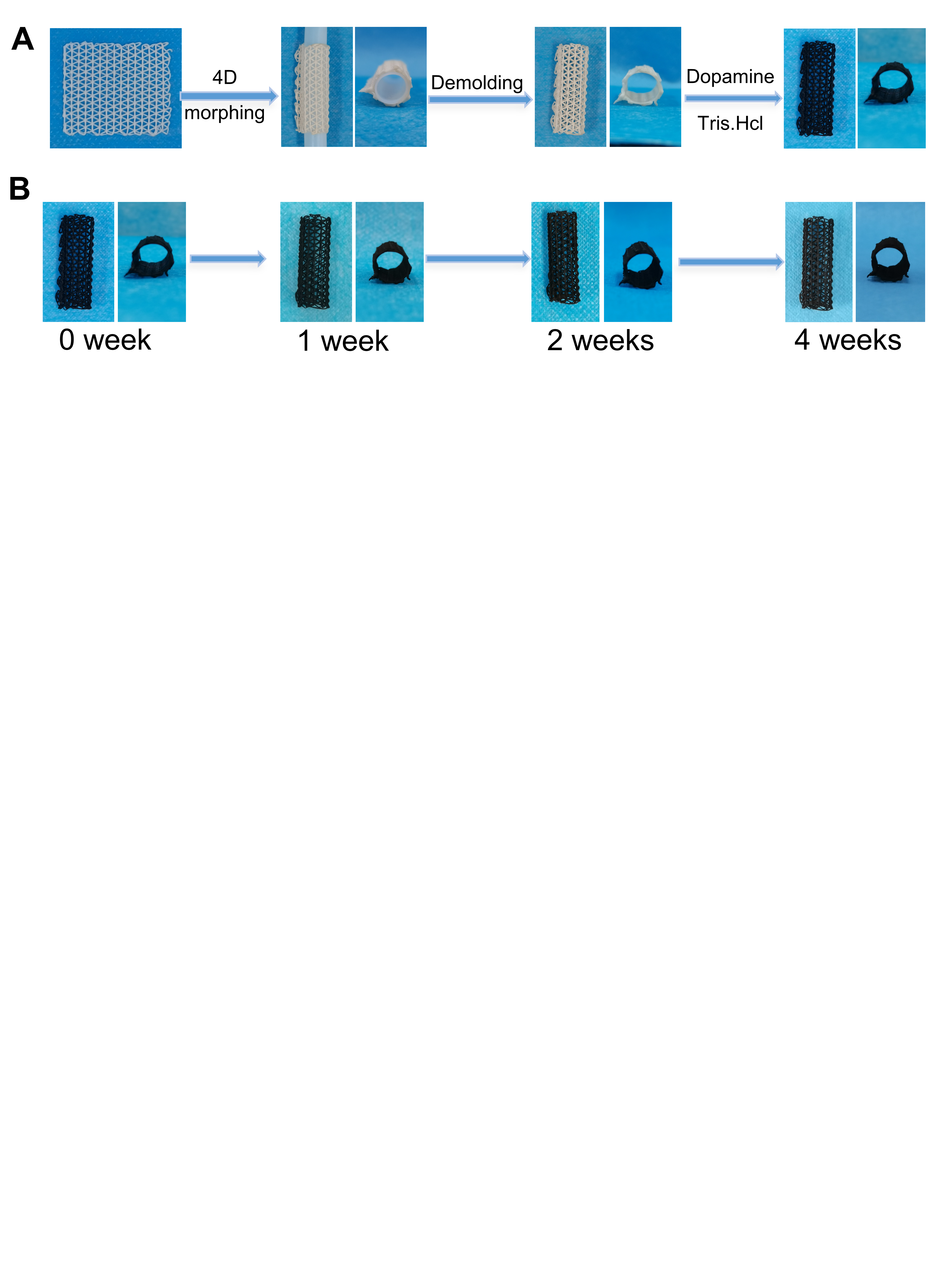

Supplement: rbae059_Supplementary_Data [file rbae059_supplementary_data.zip › Figure S5.tif]

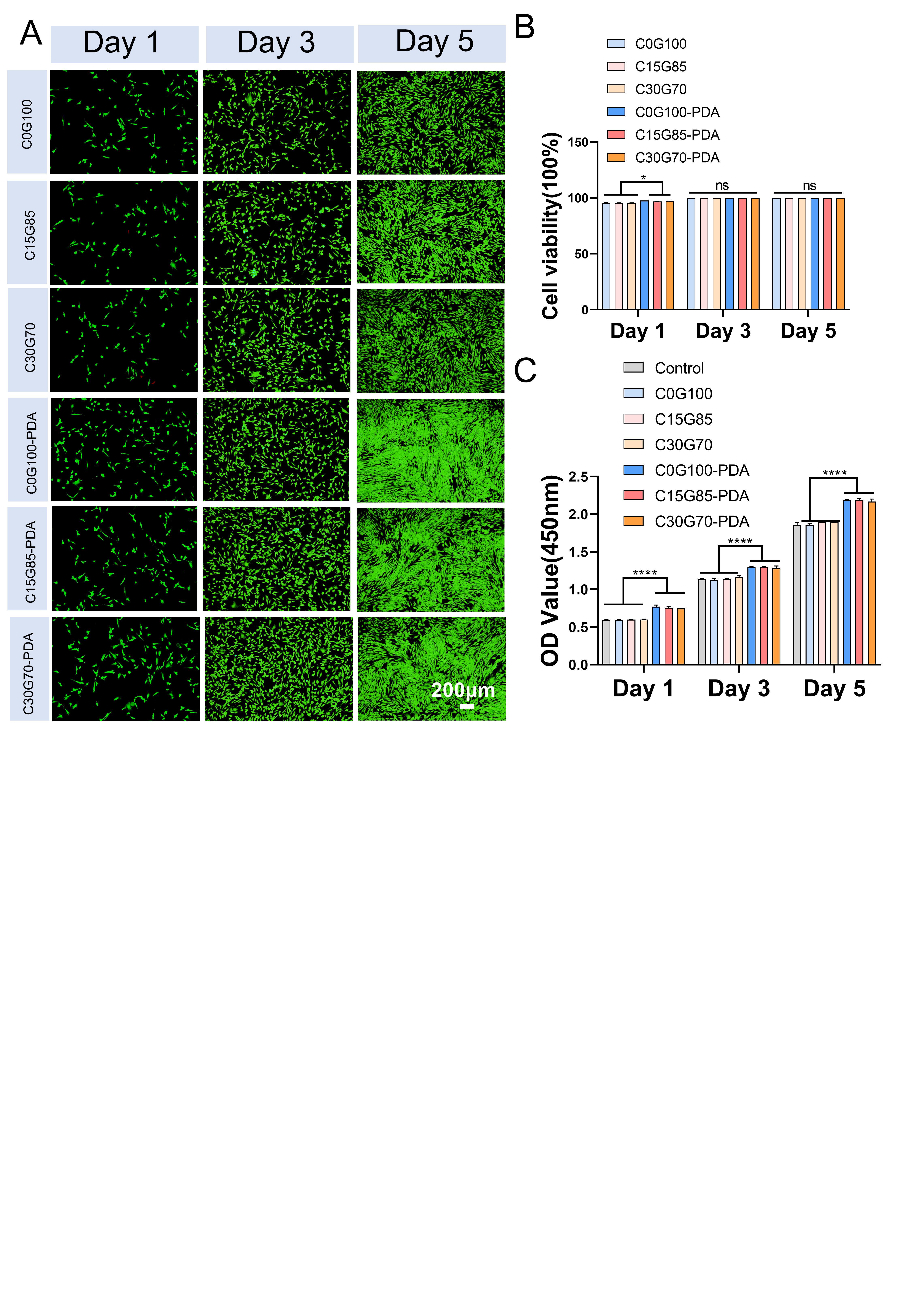

Supplement: rbae059_Supplementary_Data [file rbae059_supplementary_data.zip › Figure S6.tif]

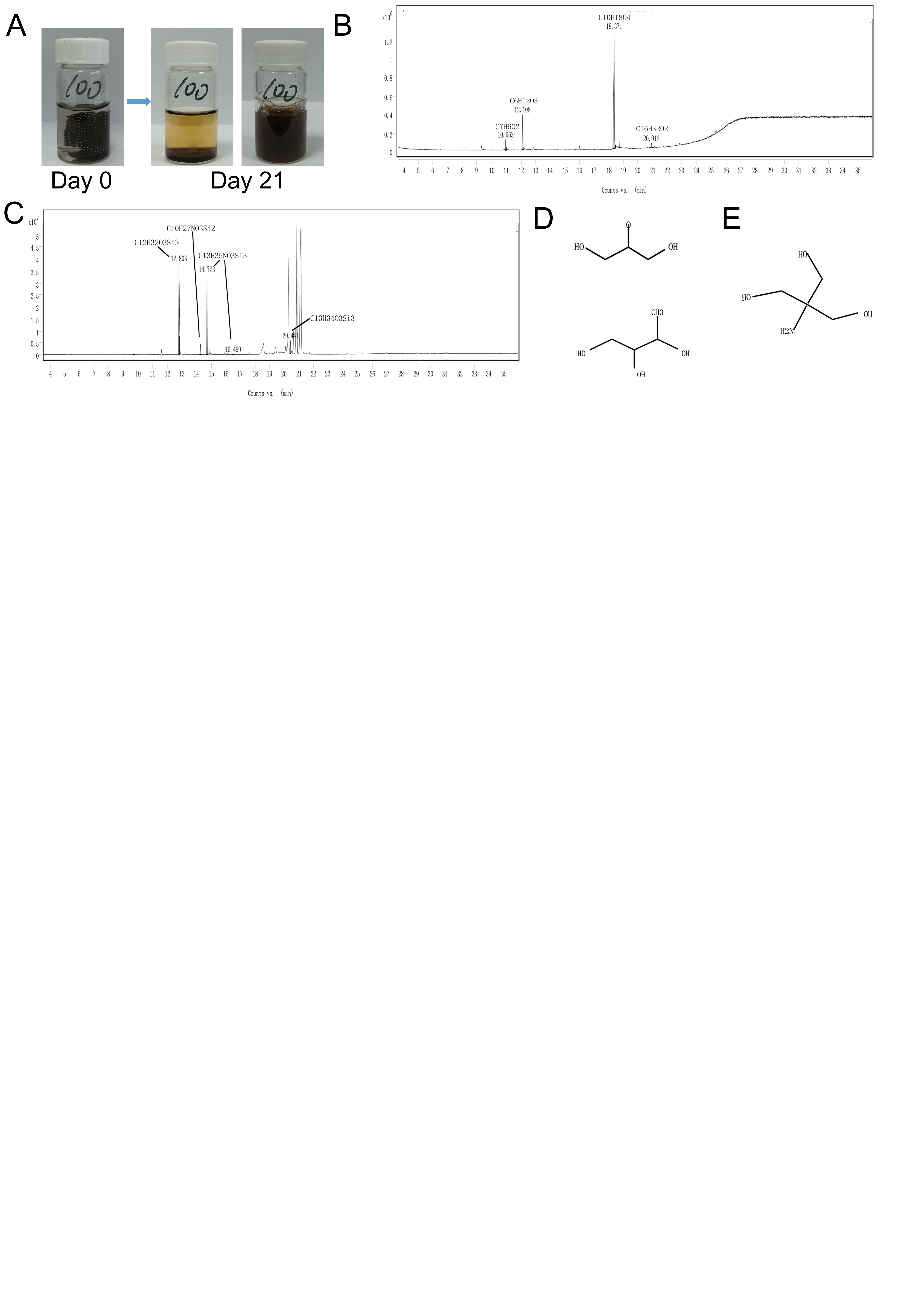

Supplement: rbae059_Supplementary_Data [file rbae059_supplementary_data.zip › Figure S7.tif]

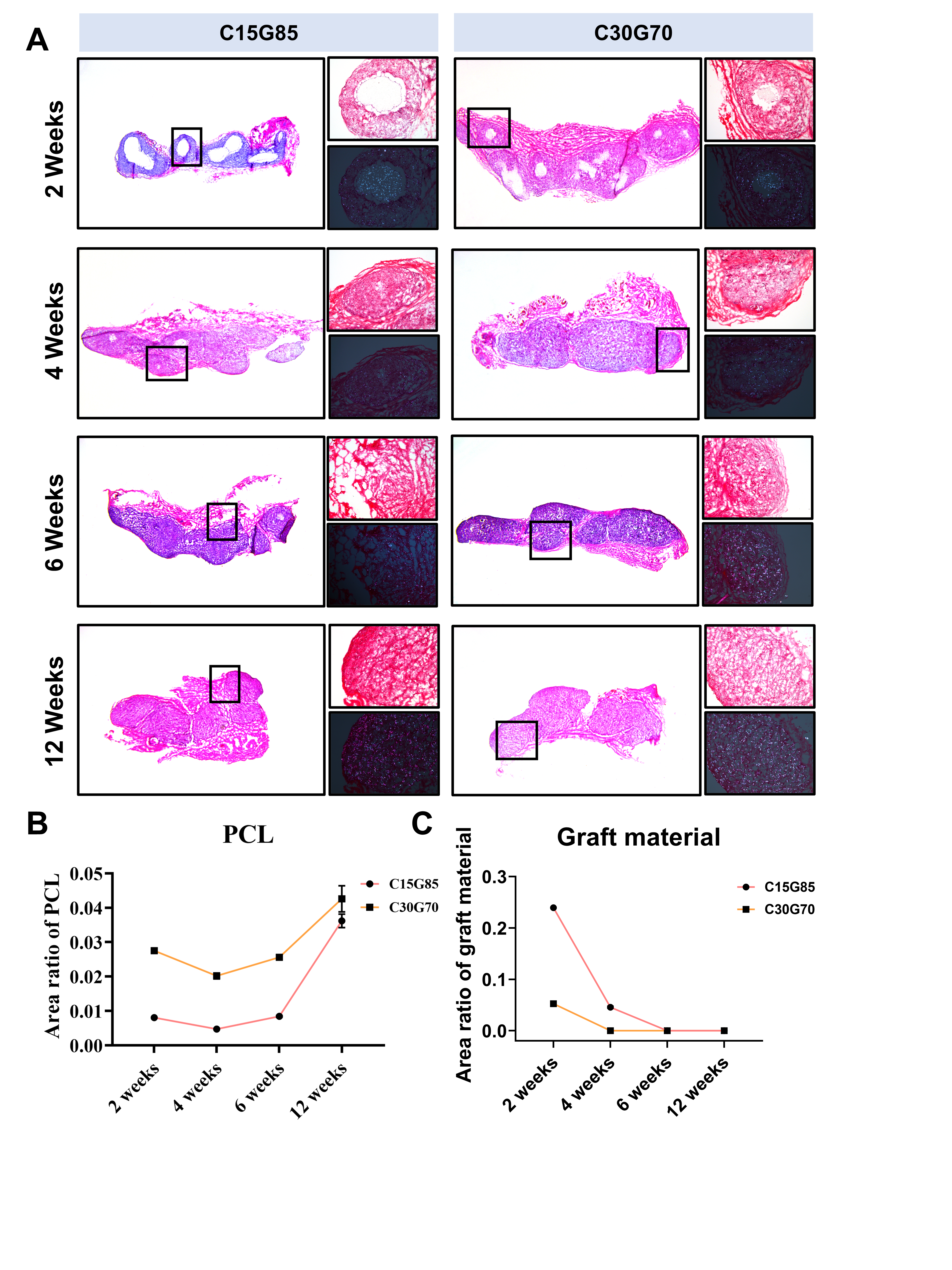

Supplement: rbae059_Supplementary_Data [file rbae059_supplementary_data.zip › Figure S8.tif]

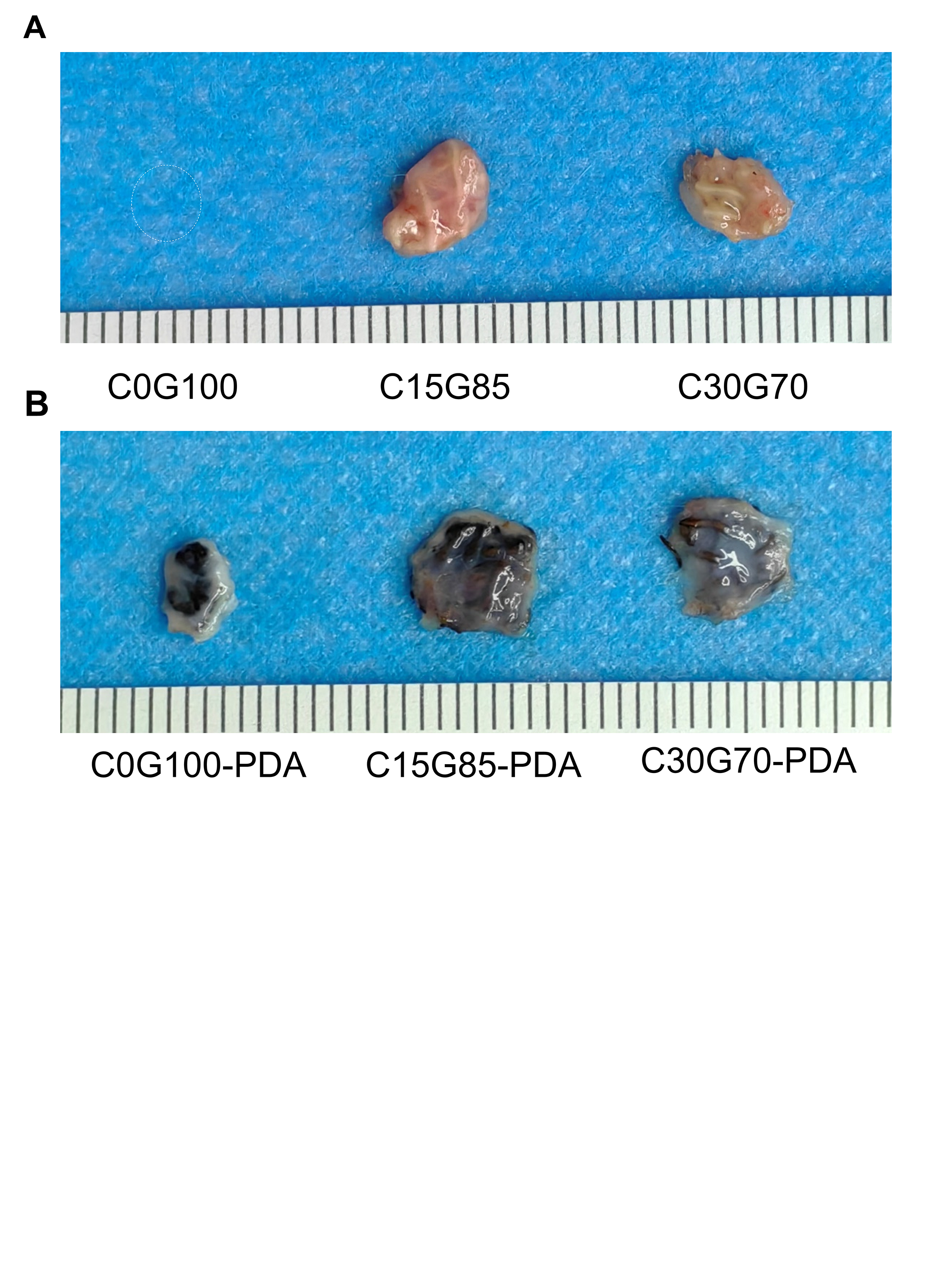

Supplement: rbae059_Supplementary_Data [file rbae059_supplementary_data.zip › Figure S9.tif]

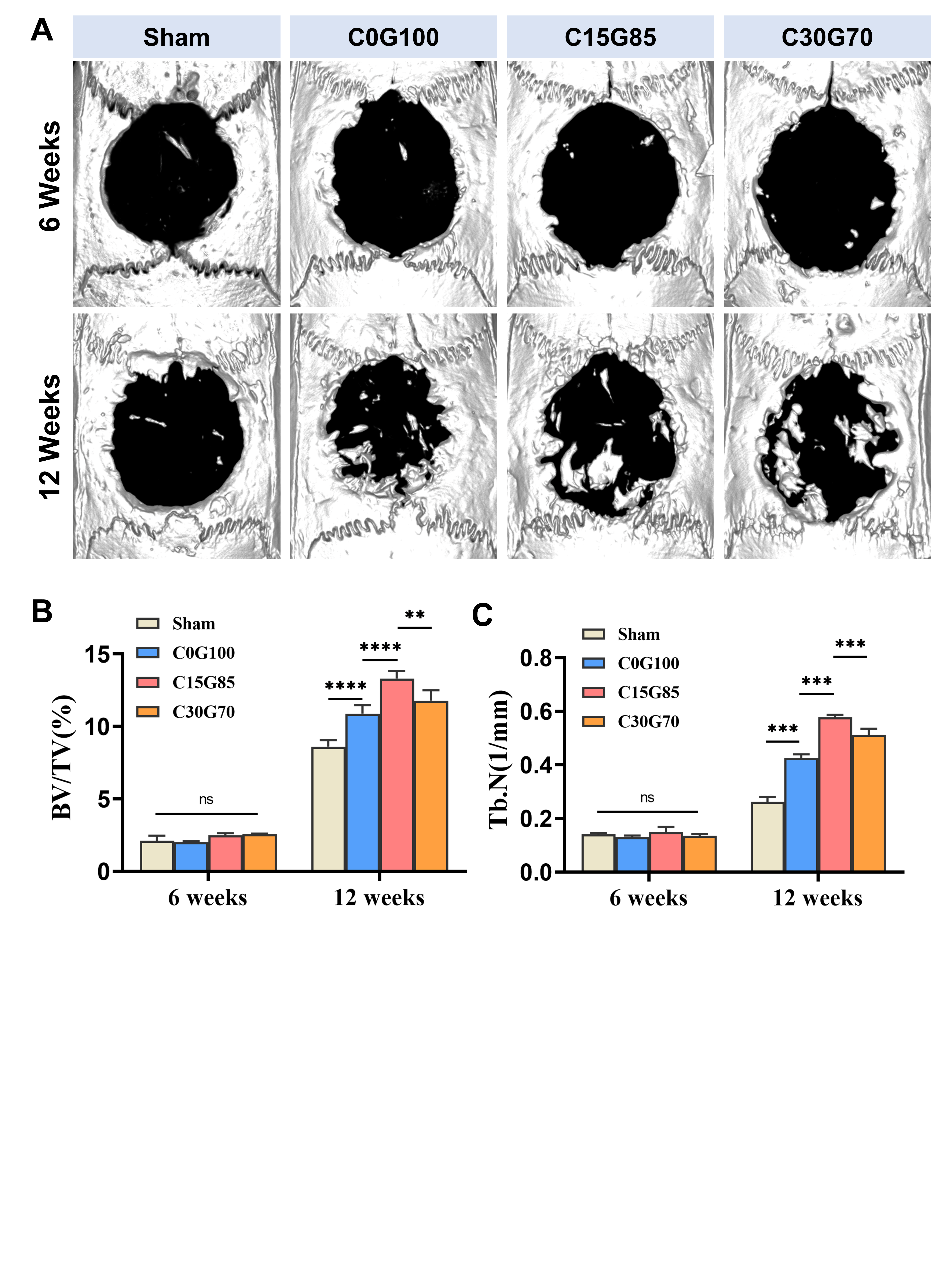

Supplement: rbae059_Supplementary_Data [file rbae059_supplementary_data.zip › Figure S10.tif]

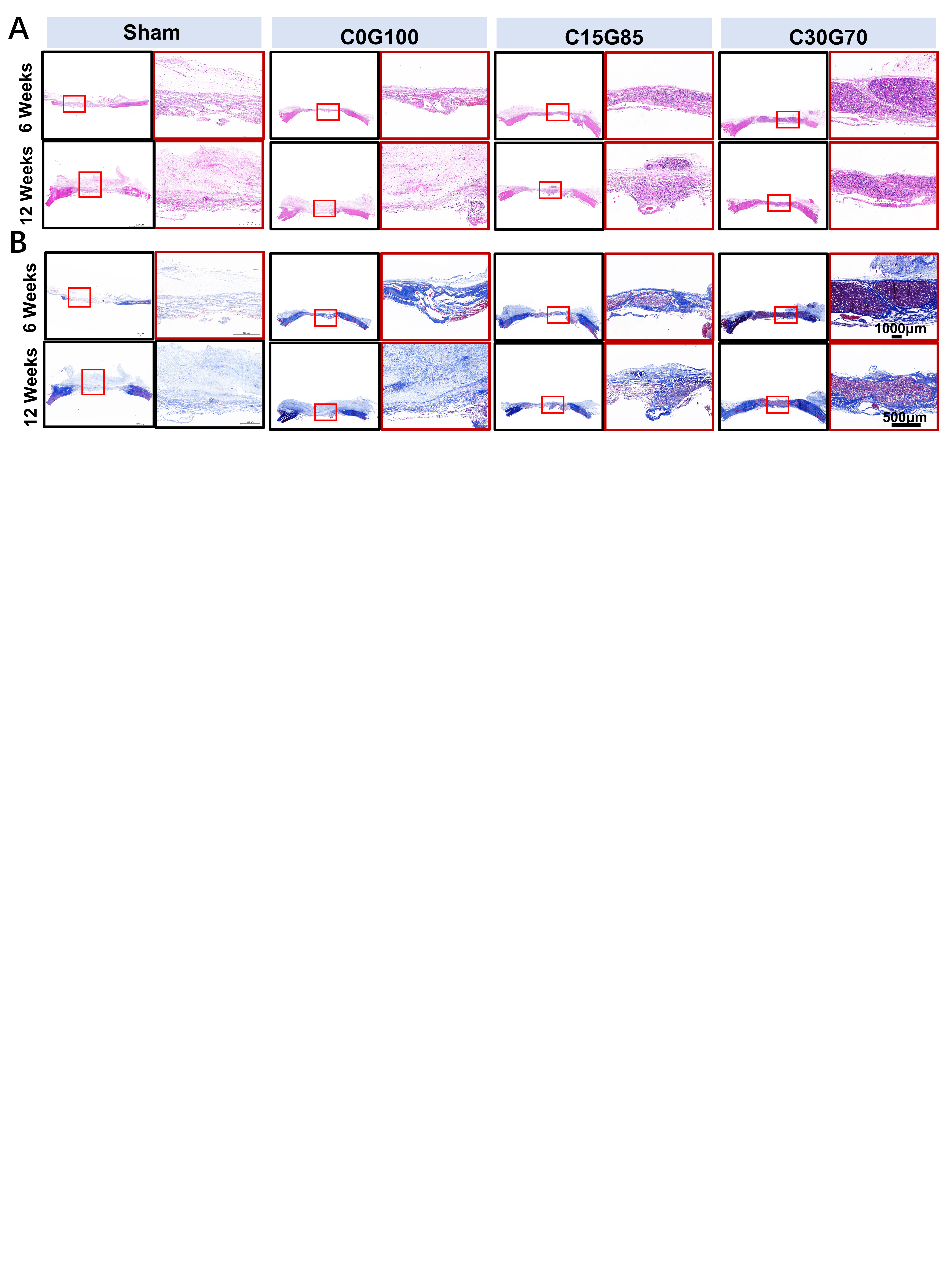

Supplement: rbae059_Supplementary_Data [file rbae059_supplementary_data.zip › Figure S11.tif]

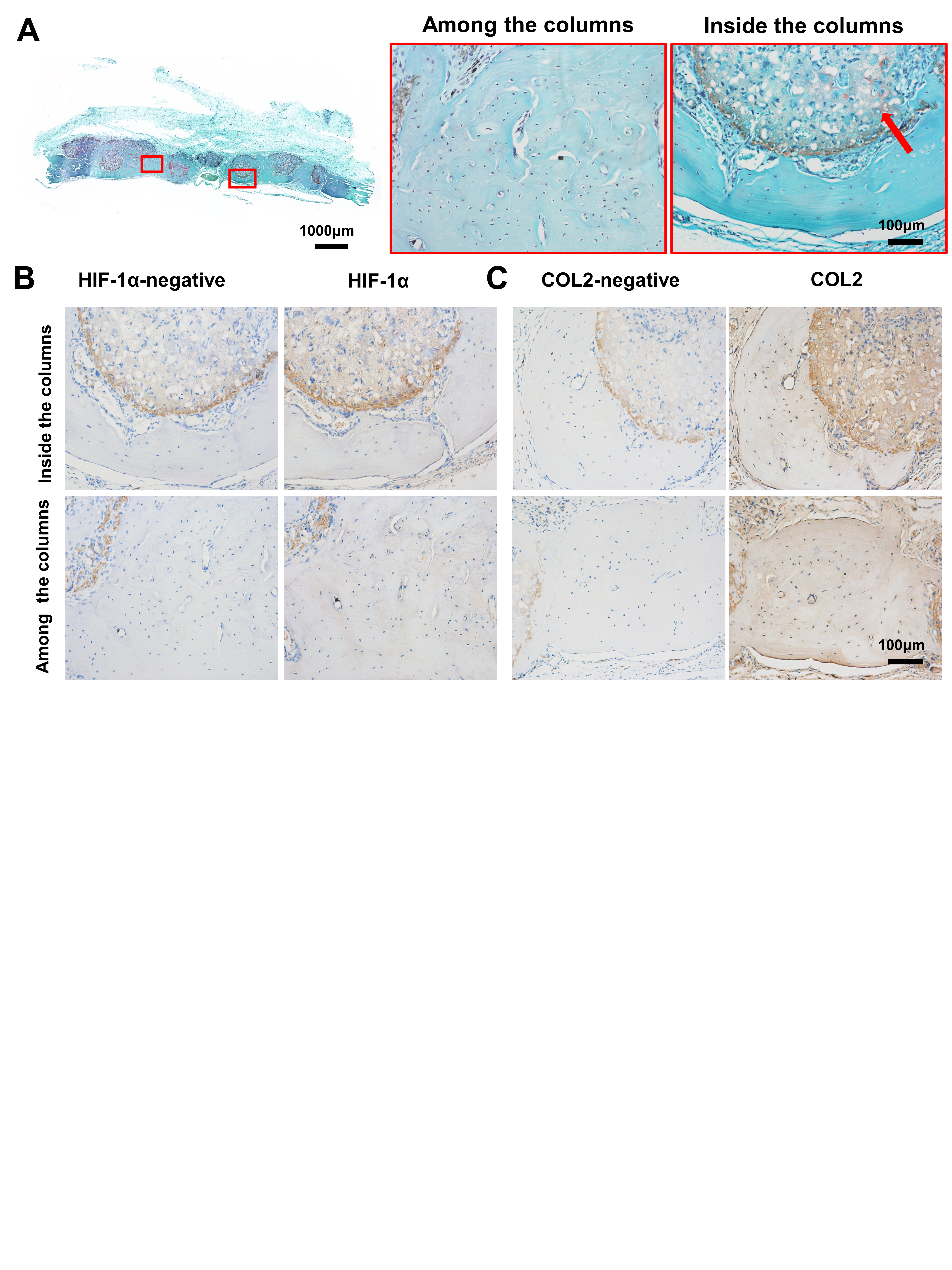

Supplement: rbae059_Supplementary_Data [file rbae059_supplementary_data.zip › Figure S12.tif]
