# Supplementary material for: Mussel inspired 3D elastomer enabled rapid calvarial bone regeneration through recruiting more osteoprogenitors from the dura mater
Source: Regen Biomater. 2024 May 22;11:rbae059. doi: 10.1093/rb/rbae059 (PMC11193312; doi:10.1093/rb/rbae059)
Supplement: rbae059_Supplementary_Data [file rbae059_supplementary_data.zip › Supplementary data.doc]

**Supporting Information**

Mussel inspired 3D elastomer enabled rapid calvarial bone regeneration through recruiting more osteoprogenitors from the dura mater

Figure S1. 3D elastomer with PDA coating in the form of GBR membrane couldn’t promote bone regeneration of 8 mm calvarial bone defect effectively. (A) 3D reconstruction of the defect area of 4D-PDA group at 12 weeks. (B) HE staining of the defect area of 4D-PDA group at 12 weeks. 4D-PDA: The PDA-coated 4D-morphing membrane composed of 3D-Printing PGS/PCL construct and electrospun PGS membrane. (The materials and methods have been described in our previous research [1]).

Figure S2. SEM examination showed microstructures of 3D porous elastomer through (A) interfacial viewing and (B) transverse viewing x at different magnifications.

Figure S3. (A) Quantitative comparison of tensile moduli of 3D porous elastomer (n = 3). (B) Quantitative comparison of compressive moduli of 3D porous elastomer (n = 3).

Figure S4. (A) 4D-morphing of 3D porous elastomers. (B) 3D graft (6.0 cm in diameter) was stably fixed to defect (5.8 cm in diameter) of 3D printed human skull model without metal plates.

Figure S5. The shape of the C15G85-PDA membrane remained stable over time. (A) The sheet material of C15G85 was shaped into a tubular material and was coated with PDA. (B) The shape of C15G85-PDA material at 0, 1, 2 and 4 weeks.

Figure S6. Biocompatibility evaluation of different groups. (A) Live/Dead staining (Merge) of rat BMSCs co-cultured with each graft for 1, 3, and 5 days. The live cells emitted green fluorescence, while the dead cells emitted red fluorescence. (B) Quantitative comparison of the green fluorescence intensity of each group (n=3). (C) Cell proliferation of rat BMSCs detected by CCK8 after 1, 3, and 5 days (n=3).

Figure S7. The biodegradability component of the PCL/PGS/PDA grafts. (A) The gross tumor morphology before and after degradation. (B) Gas chromatography-mass spectrometry of the degradation solution. (C) Gas chromatography-mass spectrometry of the silanized derivative of degradation solution. (D) Glycerolipids: C3H8O3 and C4H10O3. (E) Amines: C4H11NO3.

Figure S8. Different ratios of PCL and PGS resulted in different degradation patterns (A) HE staining visualized degradation patterns of critical bone defects in the C15G85 and C30G70 group at 2, 4, 6 and 12 weeks. (B-C) Quantitative comparison of remaining area of PCL and the graft material (n = 3). (The tissue panoramas were obtained by stereo microscope and the locally enlarged images of the tissue were obtained by polarized light microscopy) Data is presented as Mean ± SD. ns, *P* > 0.05; **P* < 0.05; ***P* < 0.01; ****P* < 0.001; and *****P* < 0.0001.

Figure S9. The gross morphology of the subcutaneous implantation model after 12 weeks. (A) The gross morphology of C0G100, C15G85 and C30G70 graft. (B) The gross morphology of C0G100-PDA, C15G85-PDA and C30G70-PDA graft.

 Figure S10. 3D elastomer without PDA couldn’t promote bone regeneration effectively. (A) 3D reconstruction of the defect area of C0G100, C15G85 and C30G70 group at 6 and 12 weeks. (B–C) Quantitative analysis of BV/TV and Tb.N of the different groups at 6 and 12 weeks (n = 5). Data is presented as Mean ± SD. ns, *P* > 0.05; **P* < 0.05; ***P* < 0.01; ****P* < 0.001; and *****P* < 0.0001.

Figure S11. HE staining (A) and Masson’s trichrome staining (B) showed the repair of critical bone defects in the C0G100, C15G85 and C30G70 group at 6 and 12 weeks (n = 5).

Figure S12. New bone developed in two ways: intramembranous ossification and endochondral ossification. (A) Safranin O/fast green staining of the newly formed bone at 6 weeks in the C15G85-PDA group. (B-C) COL2 and HIF-1α immunohistochemical staining in the C15G85-PDA group at 6 weeks. The red arrow represents cartilage tissue.

Figure S13. *In vitro* immunomodulation effect of the grafts to the macrophages. (A) Immunofluorescent staining of the CD68, iNOS and CD206 for macrophages in different groups. (B) Quantitative analysis of iNOS+ cell proportion (n=3) (C) Quantitative analysis of CD206+ cell proportion (n=3). Data is presented as Mean ± SD. ns, *P* > 0.05; **P* < 0.05; ***P* < 0.01; ****P* < 0.001; and *****P* < 0.0001.

Figure S14. UMAP analysis of the marker genes of each cell cluster from the regenerative tissue: (A) endothelia (expressing Vwf and Cdh5), (B) fibroblasts (expressing Col3a1 and Dcn), (C) mural cells (expressing Acta2 and My19), (D)neutrophils (expressing S100a9 and G0s2), (E) B/plasma cells (expressing CD79a and Mzb1), (F) monocytic cells (expressing C1qa) and (G) T/NK cells (expressing CD3d and CD3e).

Figure S15. Identification of macrophages. (A) UMAP analysis atlas of macrophage of C15G85-PDA group at 2 weeks after implantation. (B) Specific cluster identification map of macrophage. (C) and (D)the marker genes of macrophages from the regenerative tissue.

Figure S16. UMAP analysis of the marker genes of each cell cluster of macrophages from the regenerative tissue: (A) Mrc-1-macrophage (expressing Mrc1 and C1qa), (B) IL1b-macrophage (expressing IL1b and Ptgs2), (C) Spp1—macrophage (expressing Spp1 and Acp5), (D) Ccl17—macrophage (expressing Ccl17 and Cfp), (E) cycling macrophage (expressing Mki67 and Ube2c) and (F) osteoclast (expressing Mmp9 and Ctsk).

Figure S17. UMAP analysis of the marker genes of each cell cluster of fibroblasts from the regenerative tissue: (A) Dura-Arachnoid fibroblasts (expressing Alpl, Fxyd5 and Mgp), (B) Pial fibroblasts (expressing Col18a1 and Spon1), (C) Cdhr1 fibroblasts (expressing Cdhr1 and CD55), (D) Lrrc17 fibroblasts (expressing Lrrc17 and Efemp1) and (E) Ntrk2 fibroblasts (Ntrk2 and Apoe).

Figure S18. UMAP analysis of the marker genes of each cell cluster of Fibroblast from the regenerative tissue (A) Msx1 and Msx2, (B) Focx1 and Foxc2, (C) CD200 and Runx2.

Figure S19. (A) HE staining and (B) Masson’s trichrome staining at 6 weeks showed bone repair of critical bone defects in the different groups.

Figure S20. Macrophage depletion impaired the bone repair of C15G85-PDA graft. at 4 and 12 weeks. (A) 3D reconstruction of the defect area at 4 and 12 weeks of PBS and Clodronate group. (B–C) Quantitative analysis of BV/TV and Tb.N at 4 and 12 weeks in the different groups (n = 5). (D–E) HE staining and Masson’s trichrome staining visualized the repair of critical bone defects. Data is presented as Mean ± SD. ns, *P* > 0.05; **P* < 0.05; ***P* < 0.01; ****P* < 0.001; and *****P* < 0.0001.

**Table S1.** The primary antibodies used in this experiment

| Gene | Dilution | Supplier | Catalog no. |
| --- | --- | --- | --- |
| CD68  CD68  CD206 | 1:200  1:50  1:200 | GeneTex  Abcam  Proteintech | GTX41868  ab955  187041-AP |
| iNOS | 1:200 | Proteintech | 80517-1-RR |
| CD206 | 1:200 | Proteintech | 60143-1-1g |
| iNOS | 1:200 | GeneTex | GTX17504 |
| Alpl | 1:50 | Affinity Biosciences | DF6225 |
| Mgp | 1:50 | Proteintech | 10734-1-AP |
| COL2 | 1:100 | Proteintech | 28459-1-AP |
| HIF-1 | 1:100 | GeneTex | GTX17504 |

**Table S2.** The secondary antibodies used in this experiment

| Name | Dilution | Supplier | Catalog no. |
| --- | --- | --- | --- |
| Goat Anti-Rabbit IgG H&L (Alexa Fluor® 488) preadsorbed | 1:200 | Abcam | ab150081 |
| Goat Anti-Rabbit IgG H&L (Alexa Fluor® 594) preadsorbed | 1:200 | Abcam | ab150084 |
| Goat Anti-Mouse IgG H&L (Alexa Fluor® 488) preadsorbed | 1:200 | Abcam | ab150117 |

[1]. Liu X, Chen W, Shao B, Zhang X, Wang Y, Zhang S, Wu W. Mussel patterned with 4D biodegrading elastomer durably recruits regenerative macrophages to promote regeneration of craniofacial bone. Biomaterials 2021; 276:120998
